# ZFP207 sustains pluripotency by coordinating OCT4 stability, alternative splicing and RNA export

**DOI:** 10.15252/embr.202153191

**Published:** 2022-01-17

**Authors:** Sandhya Malla, Devi Prasad Bhattarai, Paula Groza, Dario Melguizo‐Sanchis, Ionut Atanasoai, Carlos Martinez‐Gamero, Ángel‐Carlos Román, Dandan Zhu, Dung‐Fang Lee, Claudia Kutter, Francesca Aguilo

**Affiliations:** ^1^ Department of Medical Biosciences Umeå University Umeå Sweden; ^2^ Department of Molecular Biology Umeå University Umeå Sweden; ^3^ Wallenberg Centre for Molecular Medicine Umeå University Umeå Sweden; ^4^ Department of Microbiology, Tumor and Cell Biology Science for Life Laboratory Karolinska Institute Stockholm Sweden; ^5^ Department of Biochemistry, Molecular Biology and Genetics University of Extremadura Badajoz Spain; ^6^ Department of Integrative Biology and Pharmacology McGovern Medical School The University of Texas Health Science Center at Houston Houston TX USA; ^7^ Center for Precision Health School of Biomedical Informatics The University of Texas Health Science Center at Houston Houston TX USA; ^8^ The University of Texas MD Anderson Cancer Center UTHealth Graduate School of Biomedical Sciences Houston TX USA; ^9^ Center for Stem Cell and Regenerative Medicine The Brown Foundation Institute of Molecular Medicine for the Prevention of Human Diseases The University of Texas Health Science Center at Houston Houston TX USA

**Keywords:** alternative splicing, pluripotency, RNA‐binding protein, ZFP207, Zinc finger protein, Post-translational Modifications & Proteolysis, RNA Biology, Stem Cells & Regenerative Medicine

## Abstract

The pluripotent state is not solely governed by the action of the core transcription factors OCT4, SOX2, and NANOG, but also by a series of co‐transcriptional and post‐transcriptional events, including alternative splicing (AS) and the interaction of RNA‐binding proteins (RBPs) with defined subpopulations of RNAs. Zinc Finger Protein 207 (ZFP207) is an essential transcription factor for mammalian embryonic development. Here, we employ multiple functional analyses to characterize its role in mouse embryonic stem cells (ESCs). We find that ZFP207 plays a pivotal role in ESC maintenance, and silencing of *Zfp207* leads to severe neuroectodermal differentiation defects. In striking contrast to human ESCs, mouse ZFP207 does not transcriptionally regulate neuronal and stem cell‐related genes but exerts its effects by controlling AS networks and by acting as an RBP. Our study expands the role of ZFP207 in maintaining ESC identity, and underscores the functional versatility of ZFP207 in regulating neural fate commitment.

## Graphical abstract

The mammalian zinc finger‐containing protein ZFP207 controls mouse ESC pluripotency as an RNA‐binding protein, orchestrating multiple post‐transcriptional programs, highlighting a novel role for a zinc finger protein beyond its function as a transcription factor.

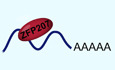

## Introduction

Mouse embryonic stem cells (ESCs) are derived from the inner cell mass of the pre‐implantation blastocyst. These cells exhibit unlimited self‐renewal capacity and, under appropriate stimuli, retain the potential to differentiate into the three germ layers (Bradley *et al*, 1984). Mouse ESCs are a useful model to study early mammalian development as their differentiation potential is more robust than that of ESC‐like cells derived from other mammals such as humans, which exhibit primed pluripotency and represent a more advanced embryonic stage (Ginis *et al*, 2004; Nichols & Smith, 2009).

Zinc finger‐containing proteins (ZFN or ZFP for human or mouse, respectively) are among the largest family of proteins, commonly containing a minimum of one zinc‐finger (ZnF) domain, which recognizes DNA sequences with high affinity. This family of transcription factors plays important roles in a variety of cellular processes including development, cellular differentiation, metabolism and oncogenesis (Cassandri *et al*, 2017). Although ZNF/ZFPs were initially classified as transcription factors, several studies have highlighted additional functions of ZNFs. For instance, it has been shown that ZFP217 could recruit the methyltransferase‐like 3 (METTL3) into an inactive complex and hence restrict N6‐methyladenosine (m^6^A) deposition on pluripotency transcripts (Aguilo *et al*, 2015; Lee *et al*, 2016). In addition, recent studies identified that ZnF domains can bind RNA, and many ZNF/ZFPs act as putative RNA‐binding proteins (RBPs) (Brannan *et al*, 2016). Indeed, analysis of quantitative global mRNA–protein interaction approaches identified ZNF207 (also termed BuGZ; Bub3 interacting GLEBS and Zinc finger domain‐containing protein) as an RBP, among other ZNFs (Baltz *et al*, 2012; Castello *et al*, 2012). ZNF207 is conserved in eukaryotes. It associates with Bub3 and with spindle microtubules to regulate chromosome alignment (Jiang *et al*, 2014, 2015; Toledo *et al*, 2014). Furthermore, both ZNF207 and Bub3 interact with the spliceosome and are required for interphase RNA splicing (Wan *et al*, 2015), yet its specific molecular role remains elusive.

In human ESCs, ZNF207 functions as a critical transcription factor by transcriptionally regulating the expression of the pluripotency factor OCT4 (Fang *et al*, 2018), thereby being implicated in the maintenance of self‐renewal and pluripotency. Likewise, ZNF207 has been shown to enhance reprogramming efficiency towards pluripotency (Toh *et al*, 2016). Alternative splicing (AS), in which splice sites in primary transcripts are differentially selected to produce structurally and functionally distinct mRNAs, plays a critical role in cell fate transitions, development, and disease (Gabut *et al*, 2011). ZNF207 undergoes AS during somatic cell reprogramming and differentiation of human ESCs, an isoform switch that seems to be required for the generation of induced pluripotent stem cells (iPSCs), and it might also be necessary to maintain ESC self‐renewal and to induce proper differentiation programs (Toh *et al*, 2016; Fang *et al*, 2018).

Here, we show that ZFP207 plays an important role in the control of mouse ESC identity by a mechanism that differs from the one observed in human ESCs. Specifically, in mouse ESCs, ZFP207 does not regulate *Oct4* transcription but increases OCT4 protein stability by disrupting ubiquitin‐dependent proteasomal degradation. In addition, depletion of *Zfp207* results in pluripotency defects and blocks neuroectodermal specification without significant changes in the transcriptome of stem cell and neural genes. ZFP207 regulates the expression of the spliceosome, and silencing of *Zfp207* leads to aberrant AS patterns. We further describe ZFP207 as a novel RNA‐binding protein (RBP), which might directly affect RNA fate. Taken together, this study uncovers the versatile species‐specific roles of ZFP207 and the link to co‐ and post‐transcriptional pathways that impact cell‐fate decisions of mouse ESCs.

## Results

### Silencing of *Zfp207* impairs proliferation and mouse ESC identity

**Figure 1:**
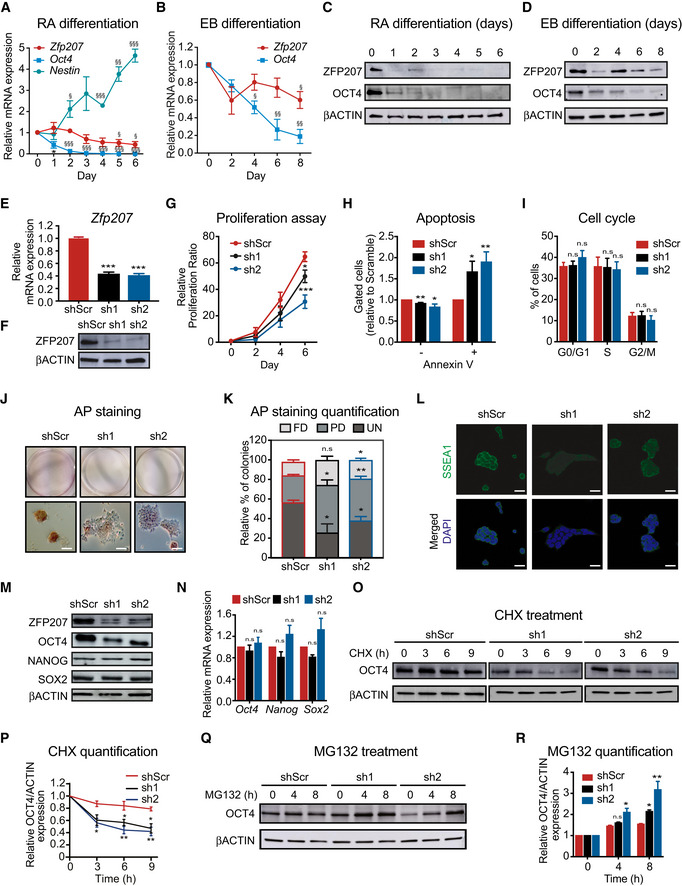
Depletion of *Zfp207* leads to growth defects of mouse ESCs A, BRT‐qPCR analysis of *Zfp207* and *Oct4* in mouse ESCs during (A) retinoic acid (RA)‐induced and (B) embryoid body (EB)‐mediated differentiation. *Nestin* was used as a neuronal differentiation marker to monitor RA‐mediated differentiation. mRNA levels are relative to the expression at day 0.C, DWestern blot of ZFP207 and OCT4 of mouse ESCs during (C) RA‐induced and (D) EB‐mediated differentiation.E, F(E) RT‐qPCR and (F) western blot to monitor the knockdown efficiency of *Zfp207* (sh1 and sh2).GCell proliferation rate of shScr, sh1, and sh2 ESCs assessed over a period of 6 days.HPercentage of live (Annexin V−) and apoptotic cells (Annexin V+) in sh1 and sh2 mouse ESCs compared to shScr.IBar chart displaying the cell cycle distribution in sh1 and sh2 mouse ESCs relative to shScr.J, K(J) AP staining of shScr and *Zfp207‐*depleted (sh1 and sh2) mouse ESCs. Scale bars, 20 µM. (K) Percentage of fully differentiated (FD), partially differentiated (PD) and undifferentiated (UN) ESC colonies in shScr, sh1 and sh2.LImmunofluorescence analysis of SSEA1 in shScr, sh1, and sh2 ESCs. DAPI was used as the nuclear marker. Scale bars, 20 μm.M, N(M) Western blot of ZFP207, OCT4, NANOG, and SOX2 in shScr, sh1 and sh2 ESCs and (N) RT‐qPCR analysis of *Oct4*, *Nanog*, and *Sox2* in shScr, sh1 and sh2 ESCs; data is relative to shScr.O, P(O) Western blot of OCT4 during a 9‐h cycloheximide (CHX) time course treatment in shScr, sh1 and sh2 ESCs. (P) Protein degradation curves were made after quantification and normalization of the bands from (O).Q, R(Q) Western blot of OCT4 during 4 and 8 h treatment with the proteasome inhibitor MG132. (R) graph bars after quantification and normalization of the bands from (Q). Data information: Data are presented as mean ± SEM or representative images of *n ≥ *3 independent biological experiments. ^§^ *P* < 0.05, ^§§^ *P* < 0.01, ^§§§^ *P* < 0.001 (D0 versus other indicated days). *P < 0.05, **P < 0.01, ***P < 0.001, n.s = no significant (shScr versus sh1 or sh2). A, B, G, H and I: unpaired Student’s *t*‐test; E, N, P and R: ordinary one‐way ANOVA. Source data are available online for this figure.

To explore the function of ZFP207 in mouse ESCs, we analyzed the expression of *Zfp207* in retinoic acid (RA)‐induced differentiation towards the neural lineage (Fig 1A) and in spontaneous differentiation of ESCs into the three germ layers by embryoid body (EB) formation (Fig 1B). Expression of the neuronal differentiation marker *Nestin* (Fig 1A) and of the pluripotency factor *Oct4* (also known as *Pou5f1*; Fig 1A and B) was used to monitor proper cell differentiation. Real‐time quantitative reverse transcription PCR (RT‐qPCR) revealed that *Zfp207* was significantly enriched in ESCs compared to differentiated cells (Fig 1A and B), and its expression levels gradually decreased along the course of differentiation, correlating with the decrease of ZFP207 and OCT4 protein levels (Fig 1C and D).

To better understand the role of ZFP207 in pluripotency and differentiation, we aimed to generate CRISPR/Cas9‐mediated knockout (KO) of *Zfp207* in mouse ESCs. We used two different strategies: (i) one single‐guide RNA (sgRNAs #1) targeting exon 3; and (ii) two distinct single‐guide RNAs (#1 and #2) targeting the region containing exon 3 and exon 9, which included the microtubule‐binding region domain of ZFP207 (Appendix Fig S1A). After picking and expanding individual clones, Western blot analysis and subsequent clone genotyping confirmed that all clones analyzed (94) displayed heterozygosity for *Zfp207* (Appendix Fig S1B–E) suggesting that homozygous deletion of ZFP207 is lethal as previously suggested (Blomen *et al,* 2015). Next, we conducted loss‐of‐function assays by using two distinct short‐hairpin RNAs (shRNAs) against *Zfp207* (thereafter referred as knockdown 1 and 2 (KD1 and KD2)), to ensure that the observed phenotype is due to *Zfp207* depletion and not due to shRNA off‐target effects. Silencing of *Zfp207* gene expression in both KD1 and KD2 ESCs was confirmed by RT‐qPCR (Fig 1E) and by Western blot (Fig 1F). Depletion of *Zfp207* led to a reduced proliferation capacity compared to ESCs transduced with scrambled shRNA (thereafter referred as control), although to a lesser degree in KD1 than in KD2 ESCs (Fig 1G; 1.3 and 2‐fold, respectively). In addition, KD1 and KD2 ESCs showed a significant 1.5‐fold increased apoptotic rate compared to control ESCs (Fig 1H) while no significant differences in the cell cycle profile were detected between the three cell lines (Fig 1I). Overall, our results indicate that ZFP207 is required for the proper proliferation of mouse ESCs.

*Zfp207*‐depleted colonies displayed the typical morphology of differentiating ESCs with flat appearance and undefined colony borders (Fig 1J). Consistently, we detected reduced metabolic activity in ESC after depletion of *Zfp207* determined by alkaline phosphatase (AP) activity assay (Fig 1J). Specifically, downregulation of *Zfp207* resulted in a significant increase in the percentage of partially differentiated colonies, whereas the percentage of undifferentiated colonies was significantly decreased compared to control ESCs (Fig 1K). Such increase in the percentage of partially and finally differentiated colonies in KD1 and KD2 ESCs is a consequence of impaired ESC function as immunofluorescence analysis revealed that silencing of *Zfp207* leads to a decrease of the pluripotency surface marker SSEA1 (Fig 1L).

Since ZFP207 regulates self‐renewal and pluripotency in human ESCs, we next analyzed the expression of the core pluripotency factors and observed that *Zfp207* KD1 and KD2 ESCs expressed lower OCT4, but not NANOG and SOX2, compared to control ESCs (Fig 1M). Interestingly, mRNA levels of the three pluripotency factors, including those of *Oct4* (Fig 1N), were unaltered upon silencing of *Zfp207*, indicating that ZFP207 could regulate the expression of OCT4 post‐transcriptionally. To test this hypothesis, we treated KD1 and KD2 as well as control ESCs with the protein synthesis inhibitor cycloheximide (CHX). Silencing of *Zfp207* led to a decrease in the half‐life of OCT4 (Fig 1O and P), suggesting that ZFP207 promotes the stability of this pluripotency factor. In addition, treatment with the proteasome inhibitor MG132 restored OCT4 protein levels in mouse ESCs depleted of *Zfp207* (Fig 1Q and R), indicating that ZFP207 interferes with the turnover of this pluripotency factor.

**Figure EV1:**
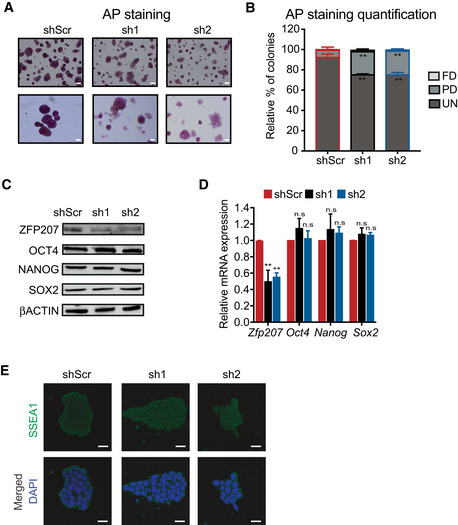
ZFP207 is dispensable in mouse ESCs maintained in ground state pluripotency A, B(A) AP staining of shScr and *Zfp207‐*depleted (sh1 and sh2) mouse ESCs cultured in 2iL. Scale bars, 20 µM. (B) Percentage of fully differentiated (FD), partially differentiated (PD) and undifferentiated (UN) ESC colonies in shScr, sh1 and sh2 cultured in 2iL.C, D(C) Western blot of ZFP207, OCT4, NANOG, and SOX2 and (D) RT‐qPCR analysis of *Zfp207,* *Oct4*, *Nanog*, and *Sox2* in shScr, sh1, and sh2 ESCs cultured in 2iL; data are relative to shScr.EImmunofluorescence analysis of SSEA1 in shScr, sh1, and sh2 ESCs cultured in 2iL. DAPI was used as the nuclear marker. Scale bars, 20 μm. Data information: Data are presented as mean ± SEM or representative images of *n* ≥ 3 independent biological experiments. **P < 0.01, n.s = no significant difference (shScr versus sh1 or sh2). B: unpaired Student’s *t*‐test; D: Ordinary one‐way ANOVA. Source data are available online for this figure.

To assess whether ZFP207 sustains self‐renewal in the naïve ground state, we cultured mouse ESCs in the presence of kinase inhibitors against MAP kinase (MEK) and glycogen synthase kinase 3β (GSK‐3β; “2i”) and leukemia inhibitory factor (LIF) (2iL medium) (Martello & Smith, 2014). Although the number of partially differentiated colonies in 2iL‐cultured ESCs increased upon silencing of *Zfp207* assessed by a loss of reactivity to AP (Fig EV1A and B), RNA and protein levels of OCT4, NANOG, and SOX2 were unaffected (Fig EV1C and D). Likewise, no changes in SSEA1 staining were observed in 2iL‐cultured ESCs upon *Zfp207* knockdown (Fig EV1E), suggesting that ZFP207 is dispensable for the maintenance of pluripotency in the ground state. Therefore, we performed the experiments in mouse ESCs cultured in conventional medium containing serum and LIF, that is, metastable ESCs, where we observed that ZFP207 contributes to stem cell identity by regulating post‐transcriptional networks.

### ZFP207 is required for proper EB differentiation

**Figure 2:**
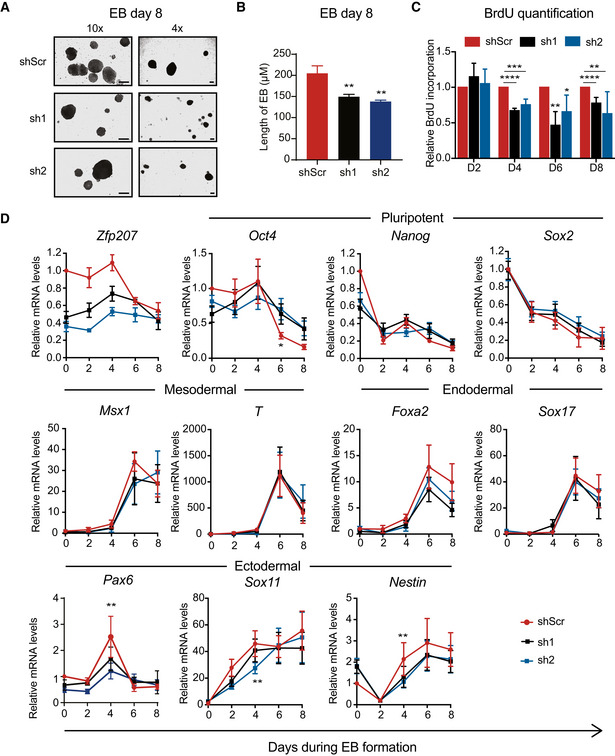
Loss of *Zfp207* results in defective differentiation A, B(A) Bright‐field images (10× (left) and 4× (right) magnification) and (B) quantification of embryoid bodies (EB) generated from shScr, sh1, and sh2 at day 8 of differentiation. Scale bars, 200 µM.CQuantification of BrdU incorporation in shScr, sh1, and sh2 ESCs at the indicated days of EB differentiation. Data are relative to shScr.DRT‐qPCR of *Zfp207*, the pluripotency genes (*Oct4*, *Nanog* and *Sox2*), the mesodermal markers (*Msx1* and *Brachyury* (*T*)), the endodermal markers (*Foxa2*, *Sox17*), and the neural‐associated genes (*Pax6*, *Sox11*, and *Nestin*) in shScr, sh1, and sh2 ESCs along the time‐course of EB‐mediated differentiation. mRNA levels are relative to the expression of shScr at day 0. Data information: Data are presented as mean ± SEM or representative images of *n ≥ 3* independent biological experiments. *P < 0.05, **P < 0.01, ***P < 0.001, ****P < 0.0001 (shScr versus sh1 or sh2). B, C: unpaired Student’s *t*‐test; D: ratio paired Student’s *t*‐test.

We next interrogated the role of ZFP207 during lineage specification by assessing the potential of KD1, KD2, and control mouse ESCs to spontaneously differentiate into EBs recapitulating early mouse embryo development. *Zfp207* KD1 and KD2 ESCs were able to form EBs (Fig 2A). Although the cells remained as solid aggregates, we observed a decrease in the size of both *Zfp207* KD1 and KD2 EBs compared to control EBs (Fig 2B), suggesting intrinsic differences during the differentiation process among the different cell lines. Given that ZFP207 influenced the apoptotic rate of ESCs (Fig 1H), we sought to investigate the mechanisms underlying the reduced size of *Zfp207* KD1 and KD2 compared to control EBs. Bromodeoxyuridine (BrdU) incorporation revealed that *Zfp207* knockdown cells were associated with a significantly reduced growth rate compared with control EBs after day 4 of differentiation (Fig 2C and Appendix Fig S2A). However, we did not observe differences in apoptosis assessed by Terminal deoxynucleotidyl transferase dUTP nick end labeling (TUNEL) staining performed along the course of differentiation or Annexin V staining of EBs at day 8 (Appendix Fig S2B–D). Altogether these results suggest that, albeit ZFP207 is critical for proper EB proliferation, its expression is not necessary for the viability of the EBs.

As expected, while *Zfp207* expression was gradually downregulated during the course of differentiation in control cells, *Zfp207* mRNA levels remained low in both KD1 and KD2 cells (Fig 2D). The expression of the pluripotent genes *Nanog* and *Sox2* decreased rapidly in all the cell lines, whereas *Oct4* decreased progressively, being its expression higher at the latest stage of the time course differentiation in EBs depleted of *Zfp207* compared to control (Fig 2D). EBs originated from the three shRNA‐infected ESCs showed normal levels of the mesodermal (*Msx1* and *Brachyury* or *T*) and endodermal (*Foxa2* and *Sox17*) lineage‐specific markers (Fig 2D). However, silencing of *Zfp207* impaired ectodermal specification as shown by decreased expression of *Pax6* and *Sox11* at day 4, and *Nestin* during the course of differentiation in *Zfp207* KD EBs compared to controls (Fig 2D and Appendix Fig S2E).

### Silencing of *Zfp207* blocks differentiation of ESCs to neural progenitor cells and subsequently to neurons

**Figure 3:**
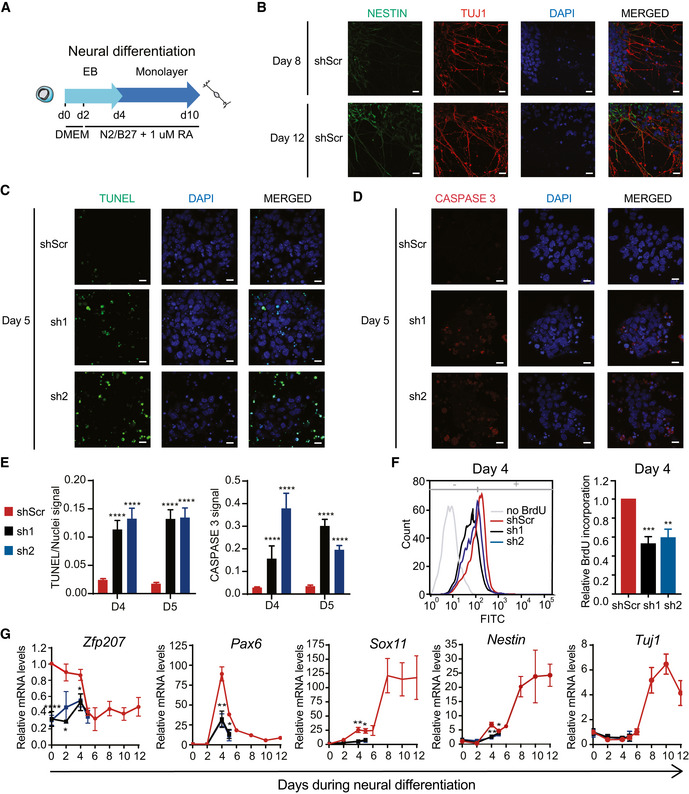
ZFP207 is essential for neural cell fate specification ASchematic depiction for the neuroectodermal‐directed differentiation. DMEM/F12 supplemented with N2B27 and retinoic acid (RA) was added after two days of EB culture.BImmunostaining of NESTIN (green) and TUJ1 (red) of neural progenitors generated from shScr on day 8 and 10 of the neuroectodermal differentiation. Nuclei were counterstained with DAPI. Scale bar, 20 μM.C–E(C) TUNEL (green), (D) CASPASE 3 (red) staining and (E) quantification of the signal in shScr, sh1, and sh2 at day 5 of neuroectodermal differentiation. Nuclei were counterstained with DAPI. Scale bar, 20 µM.FFlow cytometric profile and quantification of BrdU incorporation at day 4 of neuroectodermal differentiation.GRT‐qPCR of *Zfp207* and neural‐associated markers in shScr, sh1, and sh2 along the course of neural differentiation. mRNA levels are relative to shScr at day 0. Data information: Data are presented as mean ± SEM or representative images of *n* ≥ 3 independent biological experiments. *P < 0.05, **P < 0.01, ***P < 0.001, ****P < 0.0001 (shScr versus sh1 or sh2). E, F, and G: unpaired Student’s *t*‐test.

To investigate whether ZFP207 plays a role in neurogenesis, KD1, KD2, and control ESCs were subjected to neural differentiation (Fig 3A). Albeit the distinct cell lines did not display major morphological differences at early stages of differentiation (when growing as EBs), KD1 and KD2 underwent a complete block of neuronal differentiation potential at day five (Appendix Fig S3A), and we were not able to characterize *Zfp207* KD1 and KD2 cells at later stages as they died at day 6. However, in control cell lines, NESTIN‐ and TUJ1‐immunopositive neuronal projections appeared at day 8 and were kept in culture until day twelve (Fig 3B). Hence, we sought to also assess the viability and proliferation phenotype of *Zfp207*‐depleted mouse ESCs upon neural differentiation. Immunostaining of TUNEL and cleaved caspase 3 revealed that a large proportion of *Zfp207* knockdown cells underwent apoptosis during differentiation (Fig 3C–E, Appendix Fig S3B and C). In addition, *Zfp207* depletion dramatically decreased cell proliferation at day 4 of differentiation as measured by BrdU incorporation (Fig 3F), indicating that ZFP207 play important roles in maintaining neural stem/progenitor cell properties, including their survival and proliferative capacities.

Similar to RA‐ and EB‐mediated differentiation, *Zfp207* expression was downregulated during the course of neurogenesis in control cells, and *Zfp207* mRNA levels remained low in both KD1 and KD2 cells (Fig 3G). We also monitored progression of ESC differentiation toward neural fates by RT‐qPCR analysis of neuroectodermal markers. *Zfp207*‐depleted cell lines failed to activate the expression of *Pax6*, *Sox11*, and *Nestin* (Fig 3G and Appendix Fig S3D), suggesting a stall at early ectodermal differentiation. No defects were found in the expression of *Tuj1* at the transcriptional level as the upregulation of this neuronal marker occurred at later stages when the KD1 and KD2 cells had died (Fig 3G).

**Figure EV2:**
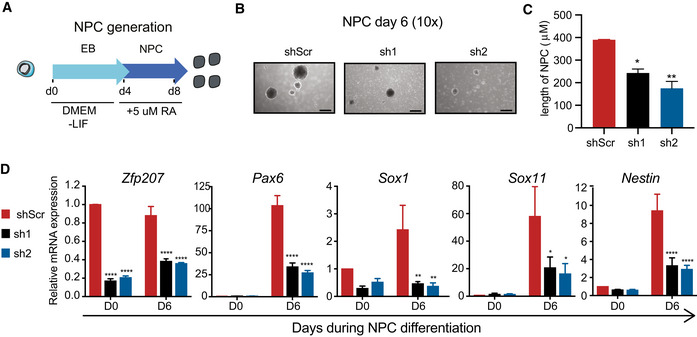
*Zfp207* knockdown ESCs cannot generate NPCs ASchematic diagram for neural progenitor cells (NPC) generation. Retinoic acid (RA) was added after four days of EB culture.B, C(B) Representative bright‐field images (10×) and (C) quantification of NPCs on day 6 of differentiation. Scale bars, 200 μm.DRT‐qPCR of *Zfp207* and neural‐associated markers in shScr, sh1 and sh2 along the course of neural progenitor (NPC) differentiation. mRNA levels are relative to shScr at day 0. Data information: Data are presented as mean ± SEM or representative images of *n* ≥ 3 independent biological experiments. *P < 0.05, **P < 0.01, ****P < 0.0001. C: Ordinary one‐way ANOVA; D: two‐way ANOVA.

We next assessed whether ZFP207 is required for early or terminal differentiation of neurons by derivation of neural progenitor cells (NPCs) from KD1, KD2, and control ESCs (Hanafiah *et al*, 2020) (Fig EV2A). Even though *Zfp207* knockdown mouse ESCs were successfully differentiated to EBs (day 4), it was immediately apparent that KD1 and KD2 NPCs were smaller and all died after day 6 (Fig EV2B and C). In addition, RT‐qPCR analysis indicated that KD1 and KD2 failed to activate the expression of ectodermal markers, again consistent with a blockade at early ectodermal differentiation upon ZFP207 deficiency (Fig EV2D).

**Figure EV3:**
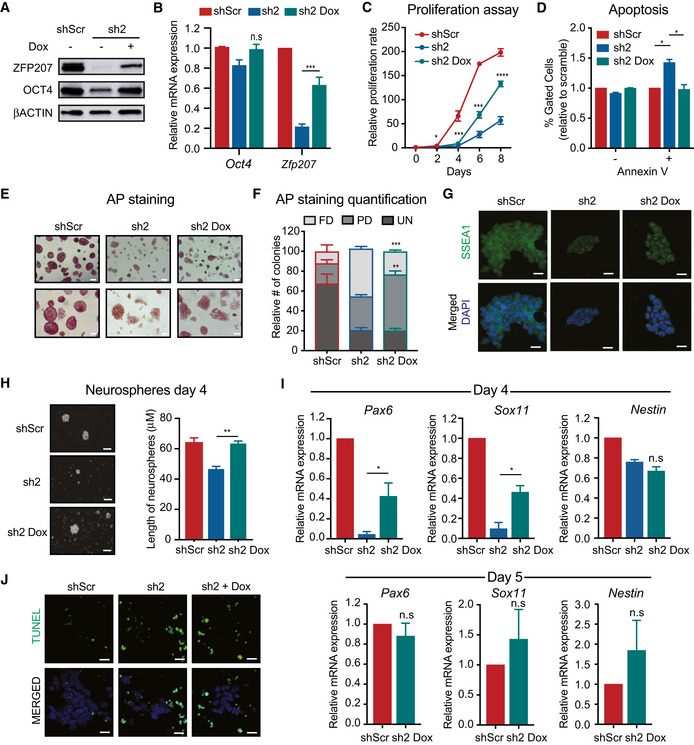
Re‐expression of *Zfp207* in tet(ON)‐ZFP207 KD2 rescues the ESC phenotype but it is not sufficient to fully differentiate tet(ON)‐ZFP207 KD2 to neurons A, B(A) Western blot of ZFP207 and OCT4 and (B) RT‐qPCR of *Zfp207 and Oct4* in the tet(ON)‐ZFP207 cell line subjected to shScr or sh2 in the absence (−) or presence (+) of doxycycline (Dox) as indicated. mRNA levels are relative to the expression in shScr.CRelative proliferation rate of tet(ON)‐ZFP207 ESCs with shScr and sh2 −/+ Dox assessed over a period of 8 days.DPercentage of live (Annexin V−) and apoptotic cells (Annexin V+) in tet(ON)‐ZFP207 ESCs with shScr and sh2 −/+ Dox.E, F(E) AP staining of shScr and sh2 −/+ Dox in tet(ON)‐ZFP207 ESCs. Scale bar, 50 µM. (F) Percentage of fully differentiated (FD), partially differentiated (PD) and undifferentiated (UN) ESC colonies in shScr and sh2 −/+ Dox treatment in tet(ON)‐ZFP207.GImmunofluorescence analysis of SSEA1 in tet(ON)‐ZFP207 ESCs with shScr and sh2 −/+ Dox. DAPI was used as the nuclear marker. Scale bars, 20 μm.HRepresentative bright‐field images (20x) and quantification of neurospheres on day 4 of differentiation. Scale bars, 200 μm.IRT‐qPCR of neural‐associated markers in tet(ON)‐ZFP207 ESCs with shScr and sh2 −/+ Dox at day 4 (upper panel) and tet(ON)‐ZFP207 ESCs with shScr and sh2 +Dox at day 5 (lower panel). mRNA levels are relative to shScr.JTUNEL (green) staining in tet(ON)‐ZFP207 ESCs with shScr and sh2 +Dox at day 5. Nuclei were counterstained with DAPI. Scale bar, 20 µm. Data information: Data are presented as mean ± SEM or representative images of *n* ≥ 3 independent biological experiments. *P < 0.05, **P < 0.01, ***P < 0.001, ****P < 0.0001, ns = no significant difference (sh2 versus sh2 Dox). B, C, D, F and I: unpaired Student’s *t*‐test. Source data are available online for this figure.

To demonstrate the specificity of the phenotype observed upon silencing of *Zfp207*, we engineered a tet(ON)‐ZFP207 cell line in which endogenous *Zfp207* can be depleted by shRNA2 and replaced with a shRNA2‐immune exogenous *Zfp207* cDNA responsive to doxycycline (Dox) treatment. As expected, Dox treatment in tet(ON)‐ZFP207 KD2 induced the expression of ZFP207 which resulted in an increase of OCT4 protein levels but not *Oct4* mRNA (Fig EV3A and B). In addition, re‐expression of ZFP207 rescued the proliferation and apoptotic defect of tet(ON)‐ZFP207 KD2 (Fig EV3C and D). Furthermore, upon addition of Dox, tet(ON)‐ZFP207 KD2 displayed a characteristic ESC‐like morphology, and the number of partially differentiated colonies was increased whereas the percentage of final differentiated colonies was significantly decreased compared to *Zfp207* KD2 ESCs without Dox induction (Fig EV3E and F). Strikingly, re‐expression of ZFP207 was not sufficient to return the normal number of undifferentiated colonies (Fig EV3E and F). Nevertheless, the levels of SSEA1 staining of tet(ON)‐ZFP207 KD2 upon Dox treatment were comparable to control ESCs (Fig EV3G), confirming that ZFP207 is critical for the maintenance of mouse ESCs.

We next investigated whether the neuroectoderm blockade was specific to the depletion of *Zfp207* by subjecting the tet(ON)‐ZFP207 cell line to neural differentiation. Of note, tet(ON)‐ZFP207 KD2 cells died at day 4; therefore, it was not possible to characterize the phenotype at later days of differentiation. However, after Dox treatment, tet(ON)‐ZFP207 KD2 regained the ability to differentiate being the size of the neurospheres similar to that of the control cells (EV3H). In addition, re‐expression of ZFP207 restored the ability to induce the expression of *Pax6*, *Sox11*, and *Nestin*, especially at day 5, where no differences in the expression of the neuroectodermal markers were detected between control and tet(ON)‐ZFP207 KD2 induced with Dox (EV3I). However, the blockade phenotype was only partially recovered as rescue cells died at later stages and displayed increase TUNEL staining (EV3J), illustrating a complex regulatory interplay of *Zfp207* isoforms occurring during neural differentiation that will be discussed later.

### ZFP207 does not transcriptionally regulate the ectodermal lineage

**Figure 4:**
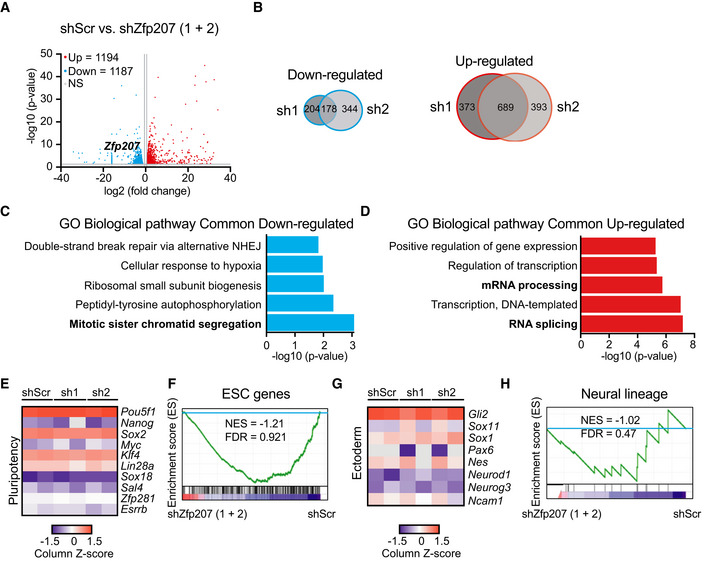
The neural transcriptome is not altered upon depletion of *Zfp207* A, B(A) Volcano plots of common differentially expressed genes in shScr and *Zfp207‐*depleted (sh1 and sh2) mouse ESCs. Upregulated (Up) and downregulated (Down) genes are indicated in red and blue, respectively (*P* < 0.05; > 1.5‐fold). *Zfp207* is depicted in black. Grey dots indicate non‐significant (NS) and < 1.5‐fold differential expressed genes. (B) Venn diagram depicting the overlap of downregulated and upregulated genes between sh1 and sh2 ESCs (FC > 1.5 and *P* < 0.05). FDR value was calculated with the Benjamini–Hochberg correction.C, DGene ontology (GO) analysis of biological processes associated with common (C) downregulated and (D) upregulated genes in *Zfp207*‐depleted ESCs (sh1 and sh2); NHEJ: Nonhomologous end joining.E–HHeatmap of column z‐scores of log2 transformed values of genes related to (E) pluripotency and (G) ectoderm in shScr, sh1, and sh2 ESCs. GSEA plots depicting the expression of (F) ESC‐related and (H) ectodermal genes in *Zfp207‐*depleted ESCs compared to shScr. High and low expression of genes is represented in red and blue color, respectively. FDR, false discovery rate; NES, normalized enrichment score.

In order to gain insight on the role of ZFP207 in ESC pluripotency, we analyzed the global transcriptome response to *Zfp207* depletion (Fig 4A). RNA‐sequencing (RNA‐seq) analysis identified 382 and 522 genes that were downregulated in KD1 and KD2 ESCs, respectively (fold change > 1.5; *P* < 0.05; Fig 4B and Dataset EV1). The differences in the number of downregulated genes between the two KDs could result in the more severe phenotype observed in cells transduced with shRNA2 compared to shRNA1 (Fig 1G). The effect on upregulation was more robust, whereby 1,062 and 1,082 genes were upregulated in KD1 and KD2 ESCs, respectively, compared to control ESCs (Fig 4B). Gene ontology (GO) analysis of biological processes of common downregulated genes revealed generic functions, which included mitotic sister chromatid segregation among other categories (Fig 4C). Strikingly, poly(A)^+^ RNA binding was among the most represented molecular function GO categories (Appendix Fig S4A). According to the reported function of ZFP207 in kinetochore–microtubule attachment (Dai *et al*, 2016), top GO categories for molecular function of common downregulated genes also included microtubule binding (Appendix Fig S4A). We validated these results by performing RT‐qPCR analysis of downregulated mitotic sister chromatid segregation genes (e.g., *Cdca8* and *Cep57l1*; Appendix Fig S4B). Upregulated genes were associated with GO biological processes related to RNA splicing and processing, and positive regulation of transcription (Fig 4D). Similar to the common downregulated genes, RNA binding, including poly(A)^+^ and mRNA binding, were among the most represented categories of GO molecular function (Appendix Fig S4C), suggesting a role of ZFP207 in controlling the expression of putative RBPs, primarily involved in RNA splicing and mRNA processing (Appendix Fig S4D).

**Figure EV4:**
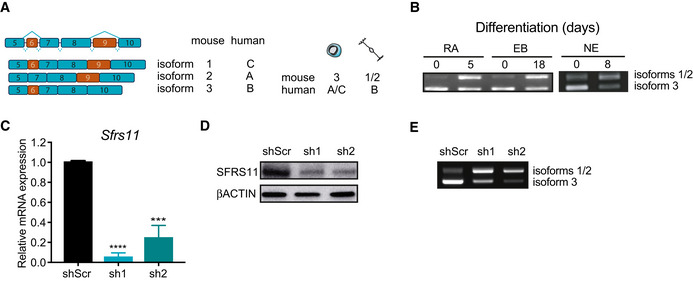
*Zfp207* undergoes alternative splicing during differentiationz A, B(A) Schema showing a segment in transcript variants with alternatively spliced exons of *Zfp207* in mouse and human (left panel). The differential switch of the splice forms between the ESC state and neural‐directed differentiation in mouse and human are also depicted (right panel). (B) RT‐PCR analysis of the AS forms of *Zfp207* at the indicated time points of retinoic acid (RA) ‐induced differentiation, embryoid body (EB) generation and neuroectodermal (NE) differentiation of mouse ESCs.C–E(C) RT‐qPCR and (D) western blot to monitor the knockdown efficiency of *Sfrs11* (sh1 and sh2). (E) RT‐PCR analysis of the AS forms of *Zfp207* upon depletion of *Sfrs11*. Data information: Data are presented as mean ± SEM or representative images of *n* ≥ 3 independent biological experiments. ***P < 0.001, ****P < 0.0001 (shScr versus sh1 or sh2). C: unpaired Student’s *t*‐test. Source data are available online for this figure.

**Figure EV5:**
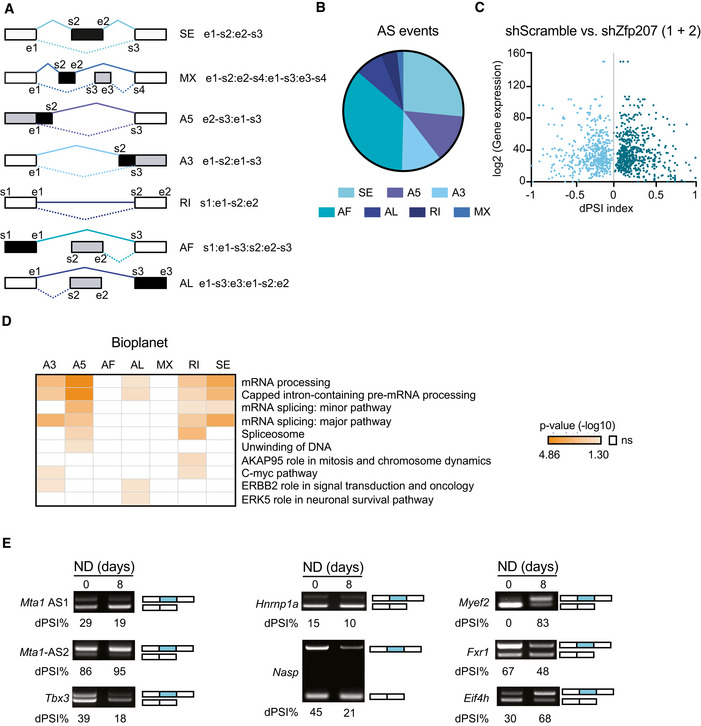
Alternative splicing switches in *Zfp207* knockdown ESCs are prevalent in differentiated cells Schema depicting the types of AS events: SE, skipped exon; MX, mutually exclusive exons; A5, alternative 5′ splice‐site; A3, alternative 3′ splice site; RI, retained intron; AF, alternative first exon; AL, alternative last exon, generated by the software SUPPA2 with the specific coordinates, including start (s) and end (e). The form of the AS event is depicted in black.Pie chart showing the AS events types distribution.Volcano plot depicting the correlation between gene expression levels and dPSI index resulted from RNA‐seq data analysis in control (scShr) and *Zfp207* depleted mouse ESCs (sh1 + sh2). Gene expression levels are presented as log2 transformed values. Differential percent spliced in index (dPSI) range is between −1 and +1.Gene ontology enrichment analysis with Bioplanet software for genes undergoing different splicing events upon *Zfp207* depletion (sh1 and sh2).Representative RT‐PCR analysis of AS events upon neural differentiation in mouse ESCs for *Mta1* (AS1 and AS2), *Tbx3*, *Hnrnp1a*, *Nasp*, *Myef2*, *Fxr1*, and *Eif4H*. The structure of each isoform is indicated (not to scale). Alternative exons are blue. PSI was quantified for each condition.

Depletion of *Zfp207* did not lead to aberrant transcriptional programs that could explain the developmental defects. Hence, there were no differences in the expression of ESC genes upon silencing of *Zfp207* (Fig 4E and F). In addition, we did not find major transcriptional differences in genes associated with the neural lineage that could reflect the stall at early ectodermal differentiation in KD1 and KD2 ESCs (Fig 4G and H). This is in striking contrast to what has been reported in human ESCs where depletion of *ZNF207* impairs neuroectodermal specification by transcriptionally regulating the expression of genes associated with the ectoderm lineage (Fang *et al*, 2018). Noteworthy, in human ESCs, the isoforms A and C of *ZNF207*, retaining the exon 9, are highly abundant compared to mouse ESCs, where the isoforms 1 and 2 of *Zfp207*, retaining the aforementioned exon 9, are highly expressed in differentiated cells (Fig EV4A and B). Hence, in both species, there is an antagonistic switch toward using different isoforms during differentiation, which could partially explain the differences observed between mouse and human ESCs. Noteworthy, *Zfp207* isoform switching occurring during differentiation could also explain why re‐expression of one isoform of *Zfp207* in the rescue experiments, that is, isoform 3, which is highly expressed in mouse ESCs, is not sufficient to fully rescue the phenotype at later stages of differentiation (Fig EV3H and I). As *ZNF207* is a downstream splicing target of SFRS11 during somatic cell reprogramming (Toh *et al*, 2016), we sought to analyze whether this splicing factor was also modulating mouse *Zfp207* splicing. Similar to what is observed in the human ortholog, depletion of *Sfrs11* increased retention of exon 9, corresponding to the isoforms 1 and 2 of *Zfp207* (Fig EV4, EV5). Therefore, SFRS11 modulates the same splicing event in mouse and human ESCs but with divergent consequences.

### ZFP207 regulates alternative splicing in mouse ESCs

**Figure 5:**
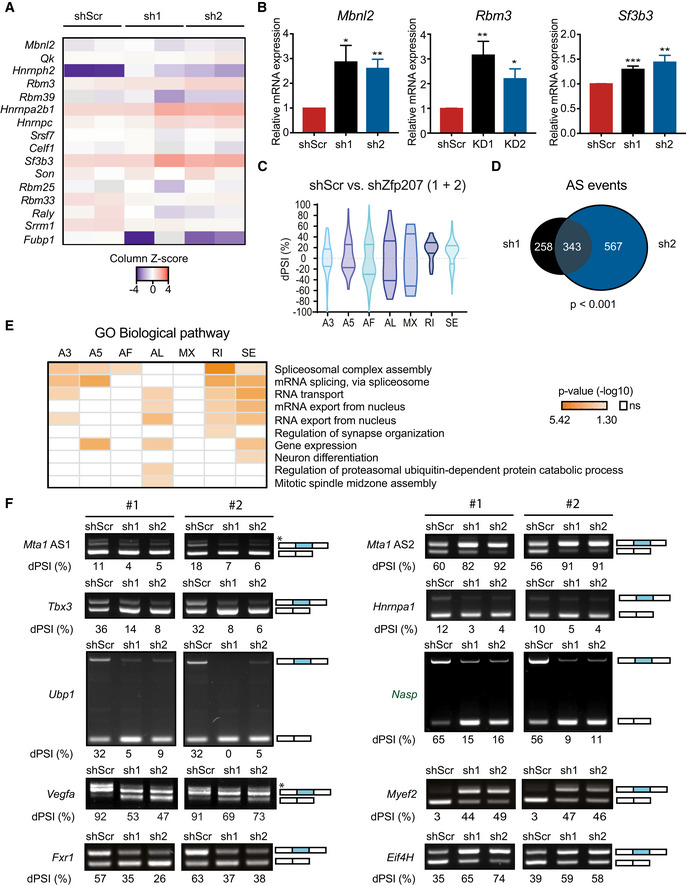
Silencing of *Zfp207* leads to AS defects AHeatmap of column z‐scores of log2 transformed values of genes involved in alternative splicing (AS) in shScr, sh1 and sh2 mouse ESCs.BRT‐qPCR analysis of *Rbm3*, *Mbnl2*, and *Sf3b3* in shScr, sh1 and sh2 ESCs. mRNA expression is relative to shScr and presented as mean ± SEM of *n* ≥ 3 independent biological experiments; *P < 0.05, **P < 0.01, ***P < 0.001 (shScr versus sh1 or sh2); unpaired Student’s *t*‐test.CViolin plot depicting statistically significant spliced in (PSI) events in each category in sh1 and sh2 related to shScr. Alternative 3′ (A3); Alternative 5′ (A5); Alternative first exon (AF); Alternative last exon (AL); Mutually exclusive exons (MX); Intron retention (RI); Exon skipping (SE). The dotted line indicates ΔPSI = 0.D, E(D) Venn diagram showing the overlap of AS in sh1 and sh2. (E) Gene ontology analysis of common genes undergoing AS in sh1 and sh2 mouse ESCs according to the type of AS event.FRT‐PCR of *Zfp207*‐regulated AS events. For *Mta1*, AS1 (alternative splicing 1) and AS2 (alternative splicing 2). #1 and #2 indicate distinct biological replicates. *Denotes an isoform that was not taken in consideration for the quantification. The structure of each isoform is indicated (not to scale). Alternative exons are blue. The percent spliced in (PSI) was quantified for each condition.

AS is a fundamental process that increases proteomic diversity in a species‐manner but not organ‐specific manner (Barbosa‐Morais *et al*, 2012), and it also plays a central role in the regulation of ESC‐specific transcriptional programs (Gabut *et al*, 2011). ZNF207 has been shown to influence pre‐mRNA splicing in cancer cells (Wan *et al*, 2015). Consistent with this observation, our GO enrichment analysis of genes upregulated upon *Zfp207* silencing revealed significant enrichment of GO terms related to RNA splicing (Figs 4D and 5A), and some randomly selected spliceosomal genes (*Mbnl2*, *Rbm3*, and *Sf3b3*) were further validated by RT‐qPCR (Fig 5B).

We therefore sought to investigate whether ZFP207 could control ESC function by a splicing‐related mechanism. To this end, reads from RNA‐seq data were mapped to exon‐splice junction’s sites in order to elucidate genome‐wide differential AS events (DSEs) (Alamancos *et al*, 2015; Trincado *et al*, 2018), including alternative 5′ and 3′ splice‐site selection (A5 or A3), alternative first (AF) and last (AL) exon selection, exons that are spliced in a mutually exclusive manner (MX) or that are skipped (SE), and changes in intron retention (RI) (Fig EV5A). Comparisons of AS isoform levels were performed in KD1 and KD2 ESCs versus control shRNA ESCs, and differences in AS isoforms were calculated as the change in percent spliced in (dPSI) (Fig 5C). Alternative first exon selection and exon skipping appeared to be the most predominant splice events (Fig EV5B). Our analysis identified 601 and 910 splicing events in KD1 and KD2 compared to control corresponding to 419 and 609 genes, respectively (false discovery rate [FDR] < 0.05; Fig 5D and Dataset EV2). Alterations in DSEs upon knockdown of *Zfp207* were not due to changes in transcription as gene expression levels were not correlated to dPSI (Fig EV5C). The overlapped AS genes were significantly enriched in functional categories associated with RNA splicing, RNA transport and export from the nucleus, and neurogenesis, among others (Figs 5E and EV5D), suggesting that splicing switches occurring upon silencing of *Zfp207* are relevant to the severe blocking defect towards the neural lineage observed in KD1 and KD2 ESCs. DSEs were validated by RT‐PCR amplifying AS exons that differ in size and band intensity were assessed in order to estimate the exon‐inclusion ratios. Specifically, we validated randomly selected ZFP207‐mediated DSEs in control, KD1, and KD2 ESCs (Fig 5F), including the chromatin regulatory cofactor *Mta1* (AS1 and AS2); the AS factors *Tbx3* and *Hnrnpa1*; the transcription factor *Ubp1*; the histone chaperone *Nasp*; the neuronal factors *Vegfa*, *Myef2* and *Fxr1*; and the translation initiation factor *eIF4H*. Furthermore, we also interrogated the aforementioned DSEs in neural differentiation, and found that the majority of the splicing switches validated in KD1 and KD2 ESCs were also prevalent in differentiated cells (Fig EV5E). Collectively, these data indicate that depletion of *Zfp207* elicits aberrant AS patterns that resemble the differentiated cell‐like pattern, and consequently influences mouse ESC identity.

### ZFP207 is a newly identified RBP

**Figure 6:**
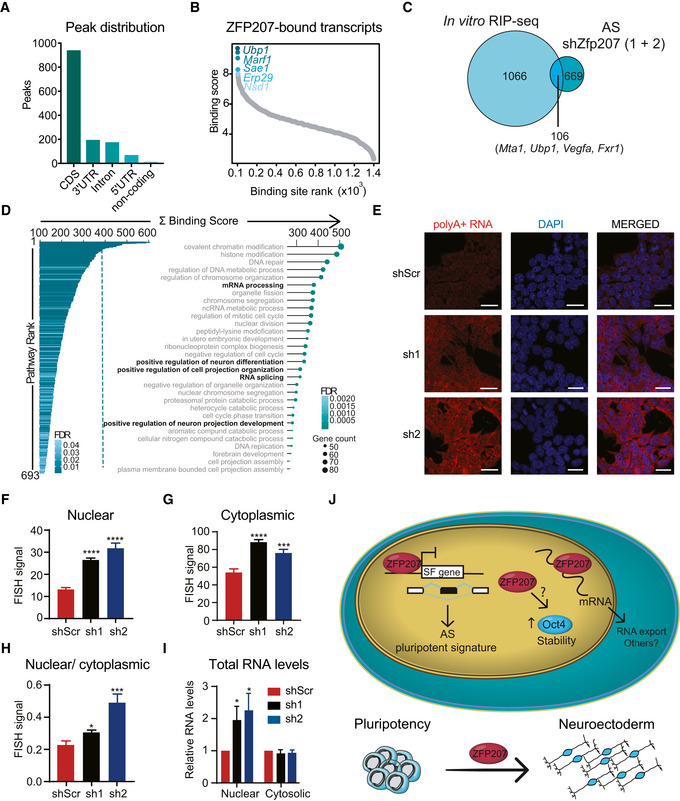
ZFP207 is a novel RBP A–D(A) Distribution of ZFP207‐binding sites across common transcript features. (B) Scatterplot depicting ranked distribution of ZFP207 binding sites. The top 5 strongest binding sites are highlighted. (C) Venn diagram of *in vitro* RIP‐seq and AS upon *Zfp207* knockdown. (D) Bar‐ and dot plots show significantly enriched GO terms for biological processes for ZFP207 bound genes. Top 30 GO terms are shown.E–I(E) FISH of poly(A)^+^ RNA distribution in shScr, sh1 and sh2 stained with 5′Cys3‐oligo dT(50). Nuclei were counterstained with DAPI. Scale bar, 20 µM. Quantification of (F) nuclear, (G) cytoplasmic, and (H) of nuclear/cytoplasmic poly(A)^+^ FISH signals. (I) Relative nuclear and cytosolic total RNA levels in sh1 and sh2 compared to shScr. Data are presented as mean ± SEM of *n* ≥ 3 independent biological experiments. *P < 0.05, ***P < 0.001, ****P < 0.0001 (shScr versus sh1 or sh2); unpaired Student’s *t*‐test.JZFP207 modulates mouse ESCs pluripotency and neuroectodermal differentiation by integrating co‐ and post‐transcriptional mechanisms.

Because ZFP207 influenced AS and it has been identified as a mRNA binder using a quantitative proteomics approach (Baltz *et al*, 2012; Castello *et al*, 2012), we assessed the innate binding propensity of ZFP207 in mouse ESCs by employing a modified *in vitro* RNA immunoprecipitation (RIP) protocol (see methods section). We identified 1,400 transcriptome‐wide binding events for ZFP207 in our assay (Dataset EV3). In total, 942 of these binding sites resided within coding regions of protein‐coding genes (Fig 6A). Since ZFP207 is located in the nucleus, we also found binding to intronic sequences (178/1,400). In addition, we detected binding to untranslated regions (UTRs) (267/1,400) and exons of noncoding transcripts (17/1,400). It is possible that there are more ZFP207‐binding events to nascent RNAs, which might have been missed since most intronic sequences are depleted by this method. The 1,400 ZFP207 binding events were distributed over 1,171 genes. Based on a fold‐change weighted by the relative expression levels (referred to as binding score), we identified the strongest binding sites within the *Ubp1*, *Marf1*, *Sae1*, *Erp29*, and *Nsd1* transcripts (Fig 6B). Surprisingly, three of the top five binding sites (*Marf1*, *Sae1*, and *Nsd1*) were found over exon–exon junctions (Appendix Fig S5A). Such binding events are difficult to envision *in vivo*, unless ZFP207 is part of the exon–exon junction complex and connects exonic ends directly.

Overlapping of the *in vitro* RIP‐seq and AS data sets revealed 13.7% of DSEs that were directly bound by ZFP207 (Fig 6C), including *Mta1*, *Ubp1*, *Vegfa*, and *Fxr1* with AS switches occurring upon silencing of *Zfp207* that were validated (Fig 5F). However, we cannot exclude that dysregulated expression of genes encoding spliceosomal factors upon depletion of *Zfp207* was a major contributor to ESC‐differential AS events. *In vivo* ZFP207 binding at randomly selected transcripts, such as the pluripotency factors *Klf4* and *Esrrb*, the RBPs *Igf2bp1* and *Fxr1*, which play a critical role in neurogenesis (Ravanidis *et al*, 2018), and the highly enriched *Sae1* transcript were validated by *in vivo* RIP followed by RT‐qPCR, thus validating our *in vitro* approach (Appendix Fig S5B and C).

In order to classify the bound genes into functional groups, we performed a pathway enrichment analysis for biological processes, and we found that the 1,171 genes fell into 693 significantly enriched pathways over the whole murine transcriptome (FDR ≤ 0.05) (Fig 6D). Interestingly, GO analysis revealed DNA repair, chromosome organization, and chromosome segregation among the most represented categories, suggesting that ZFP207 can also regulate the aforementioned pathways not just by interacting with Bub3 (Jiang *et al*, 2014; Toledo *et al*, 2014) but also by directly binding to target transcripts and influencing RNA fate. Noteworthy, GO terms that were affected upon *Zfp207* silencing such as mRNA processing, splicing, positive regulation of neuron differentiation, and neuron projection development were highly enriched. This suggests that in addition to regulating a pluripotent AS signature, ZFP207 controls the ESC state through post‐transcriptional gene regulatory mechanisms. Indeed, ZFP207‐bound transcripts fell into two major ESC gene signatures, namely, the Core and Myc modules, which are highly active in the pluripotent state, whereas the Polycomb repressive (PRC) module, operating in differentiation, was underrepresented (Appendix Fig S5D) (Kim *et al*, 2010), which is in agreement with the observation that ZFP207 regulates pluripotency by transcriptional‐independent programs. The fact that ZFP207‐bound transcripts were enriched in the Myc module, which is active in various cancers and predicts cancer outcome, may reveal additional post‐transcriptional regulatory functions of ZFP207 not just in pluripotency but also in human diseases. Depiction of the binding score of the ZFP207‐bound transcripts showed a similar binding activity pattern within the Core, Myc, and PRC modules (Appendix Fig S5E).

Because RBPs play important roles not just in mRNA splicing but also in other aspects of the post‐transcriptional mRNA fate such as nuclear export, localization, or nuclear decay (Ye & Blelloch, 2014), we analyzed the distribution of bulk poly(A)^+^ RNAs by fluorescence in situ hybridization (FISH) using an oligo(dT) probe in scramble control, KD1 and KD2 ESCs. mRNA was accumulated in nuclear poly(A)^+^ foci upon silencing of *Zfp207* (Fig 6E and F), suggesting that ZFP207 might play a role in nuclear export. Although cytoplasmic poly(A)^+^ RNA FISH signals were also brighter in *Zfp207‐*depleted cells compared to that in control cells (Fig 6E and G), the intensity of cytoplasmic poly(A)^+^ RNA pixel, and the nuclear to cytoplasmic ratios were less profound in *Zfp207* knockdown compared to control ESCs (Fig 6G and H). In addition, cellular fractionation showed a clear accumulation of nuclear RNA upon silencing of *Zfp207*, whereas no differences were detected in the amount of RNA in the cytoplasmic fractions of the distinct cell lines (Fig 6I). Although it remains possible that some mRNA will get exported from the nucleus, these results indicate that at least some particular mRNAs are nuclear retained and largely aggregate into poly(A)^+^ foci in *Zfp207* KD1 and KD2 ESCs.

In summary, we show that ZFP207 plays an important role in maintaining mouse ESC self‐renewal and neuroectodermal specification by controlling co‐ and post‐transcriptional networks. We propose a model whereby ZFP207 regulates the expression of splicing factors to promote *bona fide* splicing of a specific set of transcripts that alter cellular fate. Additionally, ZFP207 acts as an RBP to facilitate mRNA nuclear export. Hence, the versatility in regulatory capacities of ZFP207 is important for normal expression of pluripotency and neural commitment genes in order to ensure mouse ESC identity (Fig 6J).

## Discussion

Previous investigations have focused the attention on the properties of ZNF207 as a transcription factor and its function in transcriptional control (Fang *et al*, 2018). An important aspect of our findings is the observation that ZFP207 regulates mouse ESC maintenance through co‐ and post‐transcriptional mechanisms similarly to other members of the ZNF/ZFP family that can act as regulators of RNA metabolism (Hall, 2005; Razin *et al*, 2012). On the contrary to human ESCs, mouse ESCs have a naïve pluripotency with unbiased differentiation capacity, and they have proven to be an invaluable model to study development. Hence, although the human ortholog ZNF207 has been described as a critical regulator for self‐renewal and pluripotency elsewhere (Fang *et al*, 2018), we found divergent functional characteristics between both studies. These dissimilarities may reflect two distinct pluripotent states that represent mouse and human ESCs: naïve and primed, respectively (Ginis *et al*, 2004). In addition, these differences could arise from opposite AS‐shifting patterns of *Zfp207* and *ZNF207* that occur during cellular differentiation of mouse and human ESCs, respectively.

ZFP207 is essential for cellular viability in near‐haploid cells (Blomen *et al*, 2015). In line with this, we were unable to fully disrupt the *Zfp207* gene in mouse ESCs using CRISPR/Cas9 technology. Hence, we anticipate that ablation of ZFP207 is incompatible with ESC maintenance. Moreover, our results show that depletion of *Zfp207* in ESCs resulted in cell proliferation defects by triggering apoptosis, which further shifts the self‐renewal phenotype toward differentiation. Similar to human ESCs, we also found that ZFP207 controlled the expression of the pluripotency factor OCT4. However, such regulation did not occur transcriptionally as *Oct4* mRNA levels were not affected upon silencing of *Zfp207*. OCT4 is targeted for ubiquitination and degradation, and this post‐translational modification plays a critical role in OCT4 expression in ESCs (Xu *et al*, 2009). Our data suggest that ZFP207 would increase OCT4 protein stability by preventing degradation of OCT4 through the proteasome. Consistently, Jiang *et al* (2014) reported that ZFP207 functions as a chaperone that binds and stabilizes the spindle assembly checkpoint protein Bub3 by protecting Bub3 from degradation by the proteasome. It remains to be elucidated the mechanism by which ZFP207 protects OCT4 from proteasomal degradation as none of the core pluripotency factors were identified in mass spectrometric analysis of mouse ESCs using ZFP207 as bait (Jiang *et al*, 2014). However, such study identified the E3 ubiquitin ligase UBR5 as a ZFP207‐interacting partner, suggesting that ZFP207 could exert a secondary role in controlling proteasome‐mediated degradation. Strikingly, such mechanism would only operate in mouse ESCs cultured in serum medium, displaying a metastable pluripotency state, as ZFP207 does not play a critical role in the maintenance of pluripotency when ESCs are cultured in 2iL conditions where it does not modulate the expression of OCT4. This finding supports the model in which depleting a single component of the pluripotency network (such as Brd4) is not sufficient to induce perturbations in ground state pluripotency (Finley *et al*, 2018).

We assessed the function of ZFP207 during *in vitro* differentiation to the three germ layers, recapitulating the early events in embryogenesis, by performing EB‐directed differentiation. Although EB formation and growth were defective after silencing of *Zfp207*, we could conclude from these experiments that endodermal and mesoderm specification was normal whereas the neuroectodermal lineage was highly compromised. Indeed, upon neural induction, EBs depleted of *Zfp207* failed to efficiently upregulate neural markers, and neuronal differentiation appeared to stall at an early stage. In agreement with this observation, *Zfp207* knockdown ESCs were not able to generate NPCs. In contrast to human ESCs, RNA‐seq analysis revealed that ZFP207 did not drive the transcription of neuronal gene expression programs and could not explain the blockade of neuroectodermal differentiation upon *Zfp207* silencing. However, ZFP207 controlled the transcription of multiple splicing factors, which potentially could lead to aberrant AS patterns with critical functions in pluripotency. For instance, we validated the upregulated expression of *Mbnl2*, *Rbm3*, and *Sf3b3*. Accordingly, *Mbnl2* and *Rbm3* have been shown to promote differentiated‐cell‐specific AS patterns (Han *et al*, 2013; Xia *et al*, 2018; Yan *et al*, 2019). To our knowledge, no specific function of the splicing factor SF3B3 in ESCs has been described. Global profiling of the AS landscape revealed alterations of a broad number of genes. Although such splicing defects could be a consequence of an indirect effect, through dysregulation of spliceosome gene expression, we cannot exclude the possibility that ZFP207 also regulates AS in a direct manner, through interaction with spliceosome factors (Jiang *et al*, 2014). Hence, it has been shown that ZFP207 interacts with the splicing components U2AF65 and SF3a3, and transcriptomic analysis revealed splicing defects after depletion of *ZFP207* in cancer cells, although such splicing events were not thoroughly characterized (Wan *et al*, 2015). Our results expand on these prior observations reporting that ZFP207 is a major splicing regulator that plays a critical role in modulating post‐transcriptional networks that influence cell fate.

Functional categories of these AS transcripts included RNA metabolism and neuron differentiation. Interestingly, mitotic spindle midzone assembly was also among the top identified GO categories, suggesting that ZNF207 might not only promote spindle assembly by undergoing coacervation and hence, concentrating microtubules and tubulin through ZNF207 droplets (Jiang *et al*, 2015), but also by an RNA‐mediated mechanism. Randomly chosen DSE have been experimentally validated in this study. Most of them (eight out of ten) displayed a switching to a differentiated cell‐like AS pattern upon *Zfp207* knockdown, indicating that ZFP207 regulates an AS signature that controls pluripotency. In addition, several of the stem cell switches that we identified, such as *Nasp* or *Tbx3*, have been shown to be involved in cancer (Alekseev *et al*, 2011; Krstic *et al*, 2020).

By using a modified *in vitro* RIP protocol, we characterized ZFP207 as a novel RBP. ZFP207 has an annotated C2H2‐ZNFs through which RNA binding is facilitated (Brannan *et al*, 2016), and two independent interactome capture studies have retrieved ZFP207 in the mRNA‐bound proteome (Baltz *et al*, 2012; Castello *et al*, 2012). As the extent of intronic binding events that ZFP207 could potentially have inside the nucleus can be orders of magnitude higher than the ones we identified with the *in vitro* RIP, it would therefore be tempting to speculate that ZFP207 regulates AS by directly binding to specific transcripts. However, it is also plausible that the RNA‐binding activity of ZFP207 provides an additional function as we observed an accumulation of poly(A)^+^ foci in KD1 and KD2 ESCs. Interestingly, it has been shown that the poly(A)^+^ RNA foci that were accumulated upon depletion of *Zfp207* are primarily formed by exosome target mRNAs (Fan *et al*, 2018). Thus, it is possible that ZFP207 functions in the turnover of poly(A)^+^ RNA in general, and in the export of pluripotency and neuroectodermal mRNAs in ESCs. It would be interesting to determine in future studies whether the distinct *Zfp207* isoforms target a distinct set of RNAs. If that would be the case, the ESC‐specific switch in the exon 9 could drive divergent RNA‐metabolism‐based programs required for ESC self‐renewal and pluripotency.

Albeit the function of most ZNFs/ZFPs is still poorly understood, a group of proteins of this large family has been associated with the functionality of the nervous system (Al‐Naama *et al*, 2020). Hence, the Zic family of ZNPs is involved in the differentiation of neuronal progenitors in the medial forebrain and the cerebellum, whereas the Ikaros family has a similar function in the striatal medium spiny neurons (Inoue *et al*, 2008; Alsio *et al*, 2013). Similarly, ZNF367 has been shown to modulate neuroblast proliferation and neuroectodermal differentiation, suggesting a role in M phase exit or in the spindle checkpoint control prior to anaphase (Naef *et al*, 2018). Many of these ZNFs/ZFPs exert their function through RNA binding. For instance, midlife crisis (Mdlc) is required for the maintenance of neuronal differentiation in Drosophila, a mechanism that is dependent on the CCCH zinc‐finger domain and involves splicing (Carney *et al*, 2013). Another example is Unkempt, which modulates RNA trafficking to control early morphology of murine neurons (Murn *et al*, 2015). Here, we illustrate an additional ZNF/ZFP with a critical RNA‐mediated function in neuroectoderm specification, suggesting that post‐transcriptional regulation might be a common feature shared by a subgroup of ZNFs/ZFPs.

This study contributes to a general and better understanding of the coordinated control of gene expression mediated by ZNPs/ZNFs during neuronal differentiation. In addition, it sheds light on the function of ZFP207 and on our understanding of how co‐ and post‐transcriptional programs ensure gene expression to maintain the ESC state. Given that ZFP207 binds both DNA and RNA, the relative contributions between transcriptional and post‐transcriptional networks cannot be discriminated. Hence, we cannot exclude the possibility that ZFP207 may also regulate self‐renewal transcriptionally, that is, by regulating the expression of splicing factors. Thus, ZFP207 provides an extra layer of control to fine‐tune the balance between self‐renewal and neuronal lineage commitment in mouse ESCs.

## Materials and Methods

### Constructs

cDNA of *Zfp207* isoform 3 from mouse ESCs was generated (RevertAid^®^ First Strand cDNA Synthesis Kit) and cloned into pGEM^®^‐T easy vector (Promega), and ultimately subcloned into the p3XFLAG‐CMV‐8 (Sigma‐Aldrich) vector using *Hind*III and *Bgl*II as restriction enzymes. To generate the rescue construct, p3XFLAG‐CMV‐8‐*Zfp207* was used to mutate the region of *Zfp207* targeted by shRNA2 by using the Quikchange Lightning multisite‐directed mutagenesis kit (Agilent). DNA sequencing confirmed the single nucleotide mutations. The mutated *Zfp207* was then amplified with specific primers harboring restrictions’ sites extensions for *Esp3*I and *Xho*I and subcloned in the Lenti‐iCas9 neo vector (Addgene 85400), which was digested with the same restriction enzymes, resulting in the loss of the iCas9 region. The resulting construct was thereafter referred to as tet(ON)‐ZFP207. All primers used for cloning purposes are described in the Appendix Table S1.

### Antibodies

The following commercially available antibodies were used at the indicated concentrations for western blot: Anti‐ZFP207 (Santa Cruz Biotechnology, sc‐271943, 1:500), Anti–β‐ACTIN (Sigma‐Aldrich, A5441, 1:2,500), Anti‐OCT3/4 (Santa Cruz Biotechnology, sc8628, 1:2,500), Anti‐Nanog (Santa Cruz Biotechnology, sc‐374001, 1:1,000), Anti‐Sox2 (Santa Cruz Biotechnology, sc‐398254, 1:1,000), Anti‐SFRS11 antibody (Abcam, ab196801, 1:2,000), Goat Anti‐Rabbit IgG H&L (HRP) (Abcam, ab6721, 1:5,000), Goat Anti‐Mouse IgG H&L (HRP) (Abcam, 1:1,000, ab6789), Rabbit Anti‐Goat IgG H&L (HRP) (Abcam, 1:5,000, ab6741), and Rabbit Anti‐ goat IgG (HRP) (Abcam, ab6771, 1:5,000), Anti‐Flag (Sigma, F3165, 1:1,000). In all western blots, β‐ACTIN was used as a loading control (Sigma, A5441, 1:2,500). For IF staining, we used Anti‐SSEA1 (Invitrogen, MA5‐17042, 1:250), Anti‐Nestin (Abcam, ab81462, 1:50), Anti‐Tuj1 (Abcam, ab18207, 1:200), Anti‐Caspase 3 Antibody, active (cleaved) form (MERK, AB3623, 1:100), Goat anti‐mouse IgG AF488 (Invitrogen, A11029, 1:1,000), Goat anti‐rabbit IgG AF568 (Invitrogen, A11011, 1:1,000), Anti‐Mouse IgG HRP (Abcam, ab6789, 1:1,000), and Donkey Anti‐Goat IgG AF594 (Invitrogen, A11058, 1:250).

### Cell culture

CCE murine ESCs were cultured on 0.1% gelatin‐coated tissue culture plates under feeder free culture conditions at 37°C with 5% CO_2_ in a humidified incubator. The media composition consists of Dulbecco’s modified Eagle’s medium (DMEM) high glucose, 15% fetal bovine serum (FBS; Gibco), 1% MEM non‐essential amino acids (Sigma‐Aldrich), 0.1 mM of 2‐β mercaptoethanol, 1% l‐glutamine (Hyclone) and 1% penicillin/streptomycin (Gibco) and Leukemia inhibitory factor (LIF; R&D systems). ESCs in the 2iL medium were grown in a 1:1 mix of DMEM/F12 (Gibco) and Neurobasal medium (Gibco) with 1× N2‐Supplement (Gibco, Cat. 17502048), 1× B27 minus insulin (Gibco, Cat. A1895601), 0.05% BSA (Gibco), Leukemia inhibitory factor (LIF; R&D systems), 2 mM Glutamine (Gibco), 1% penicillin/streptomycin (Gibco), 1 μM PD03259010 (Mek Inhibitor; MedChemExpress), 3 μM CHIR99021 (GSK3b Inhibitor; Stem cell technology), 1.4 × 10^−4^ M Monothioglycerol (Sigma) at a density of 1 × 10^5^ per well. The media was replaced every 48 h.

### Lentiviruses production and generation of *Zfp207* KD mouse ESCs

Lentiviral particles were generated by transfecting HEK‐293T with the following lentiviral plasmids: (i) pLKO.1‐Puro containing shRNA1 and shRNA2 against *Zfp207* (Appendix Table S1); (ii) pLKO.1‐Puro containing shRNA1, shRNA2 and shRNA3 against *Sfrs11* (Appendix Table S1); and (iii) tet(ON)‐ZFP207 with the packaging vector pCMV‐dR8.2 and the helper plasmid pCMV‐VSV‐G using jet‐PEI (Polyplus) as per the manufacturer’s instructions. Lentiviral supernatants were collected after 48 h of incubation and concentrated using Amicon Ultra‐15 Centrifugal Filter Units (Merck). Early passage mouse ESCs were transduced with the lentiviral particles in mouse ESC media supplemented with polybrene (8 µg/ml) for 24 h. After 36 h, infected cells were treated with puromycin (2 µg/ml) for additional 6 days.

### Generation of ZFP207‐inducible ESCs

Mouse ESCs were transduced with tet(ON)‐ZFP207 with concentrated viral particles as indicated above. At 48 h upon transduction, cells were treated with Neomycin (250 μg/ml) for 6 days to obtain a stable cell line. The knockdown of endogenous *Zfp207* was done using pLKO.1 shRNA2 as described above. Infected cells were selected by addition of puromycin (2 μg/ml) into the culture media and simultaneously induced with doxycycline (250 ng/ml) during the entire experimental period.

### Generation of CRISPR/Cas9 knockout ESCs

sgRNAs were designed using the E‐CRISP online tool (http://www.e‐crisp.org/E‐CRISP/aboutpage.html) (Appendix Table S1) (Ran *et al*, 2013). All sgRNA‐Cas9 plasmids were obtained by ligation (T7 DNA ligase, Fermentas) of annealed complementary oligonucleotides of the 20‐nucleotides target sequences with the pSpCas9(BB)‐2A‐Puro (PX459) vector (Addgene plasmid #62988) digested with BbsI (BpilI) (Thermo Scientific). One day before transfection, mouse ESCs were seeded in a 12‐well plate at a density of 80,000 cells/well. A day after that, cells were transfected using Lipofectamine (Invitrogen) with 0.8 µg of Cas9 expression vector containing the corresponding sgRNAs. After 24 h, transfected cells were diluted and treated with puromycin to obtain isogenic cell clones. Isogenic cell clones were picked and expanded for 10–15 days to identify indels (insertion and deletion). KO were screened with PCR, Sanger sequencing and evaluated by western blot analysis.

### EB and RA differentiation assays

EBs were obtained by growing mouse ESCs in low‐attachment dishes in the presence of complete medium (DMEM high glucose, 15% FBS (Gibco), 1% MEM nonessential amino acids (Sigma‐Aldrich), 0.1 mM of 2‐β mercaptoethanol, 1% l‐glutamine (Hyclone), and 1% penicillin/streptomycin (Gibco) without LIF at a density of 8.8 × 10^4^ cells/cm^2^. Medium was replenished every 48 h. EBs were harvested for extraction of total RNA, whole cell extract, and nuclear protein extraction at the indicated time points. RA differentiation was performed plating mouse ESCs onto tissue culture dishes pre‐coated with 0.1% gelatin at a density of 2.1 × 10^3^ cells/cm^2^ in complete media (without LIF) supplemented with 5 μM RA (Sigma‐Aldrich) for 6 days. Differentiation media containing RA was replenished every 48 h.

### Neural differentiation assay

Differentiation toward the neuroectodermal lineage was performed as previously described (Aguilo *et al*, 2017). Briefly, 5 × 10^4^ cells/ml were plated in low‐attachment dishes in the presence of complete medium without LIF. Following EB formation for 2 days, EBs were cultured in media containing DMEM/F‐12, 0.1 mM of 2‐mercaptoethanol, N2 supplement (100×) (Gibco, Cat. 17502048), B27 supplement (50×) (Gibco, cat. 17504044), and penicillin/streptomycin (Gibco) supplemented with 1 μM of RA (Sigma‐Aldrich) for 4 days to allow neuroectodermal differentiation. On day 4, EBs were transferred onto tissue culture dishes and precoated with 0.1% gelatin, and neuroectodermal differentiation media was replenished every 48 h. Differentiated cells were harvested for total RNA, nuclear protein extraction, and immunostaining analysis at the indicated time points. Cell cultures were maintained at 37°C with 5% CO_2_ in a humidified incubator.

### Generation of neural progenitor cells

Generation of NPCs was based on EB formation as described by Hanafiah *et al* (2020). Briefly, 1 × 10^5^ cells were seeded per plate in low‐attachment dishes in the presence of a complete medium without LIF for EB formation. After 4 days, the culture media was supplemented with 5 μM of RA (Sigma‐Aldrich) for 4 days. Cells were harvested for total RNA at different time points, and media was replenished every 48 h. Cell cultures were maintained at 37°C with 5% CO_2_ in a humidified incubator.

### Cellular proliferation, cell cycle, and apoptosis assays

Cellular proliferation, cell cycle, and apoptosis assays were carried out using a Muse Cell Analyzer (Millipore, Sigma‐Aldrich) following the manufacturer’s recommendations.

### Terminal deoxynucleotidyl transferase dUTP nick end labeling (TUNEL) assay

TUNEL assay (in situ Cell Death Detection Kit, TMR red; Roche, Mannheim, Germany) for EBs and NE differentiation was performed as per manufacturer indication with slight modifications. Briefly, EBs were collected in tubes at days 4, 6, and 8 and fixed with 4% paraformaldehyde for 30 min and permeabilized with a solution containing 0.5% Triton‐100 and 0.1% Tween‐20 in 1× PBS for 1 h at RT. Thereafter, EBs were incubated in 50 µl of TUNEL staining mix solution in a humidified chamber for 1 h at 37°C in darkness. Following incubation, EBs were washed with PBS and stained with DAPI (4′,6‐diamidino‐2‐phenylindole; Invitrogen) for 5 min at RT, washed again and mounted on slides. Whole mount EBs were analyzed using the Zeiss 710 Confocal Microscope. For NE, the same protocol was followed except that for day 5, cells were grown and fixed on cover slips. Images were acquired using a Nikon microscope (ZEN lite software) and analyzed using ImageJ. For quantification of TUNEL staining, images containing at least 20–30 nuclei were only considered. For EB, images were taken simultaneously in different focal planes. Then, the total fluorescence signal was measured using a threshold setting. Thereafter, data are represented as the ratio of TUNEL to nuclei signal. All the images were exposed with the same magnitude of gain.

### BrdU incorporation assay

BrdU incorporation assay was conducted to monitor DNA replication of EBs at days 2, 4, 6, and 8 of differentiation using a FITC BrdU Flow Kit following the manufacturer’s protocol with some modifications. Briefly, the cells were incubated without or with 10 μM of BrdU (Acros Organics) for 3 h in the differentiation media. After incubation, EBs were harvested by washing with 1× PBS and fixed with cold 70% ethanol at −20°C for 4 h. After washing twice with 1× PBS, EBs were denatured in 4 M of HCl for 20 min at RT. Thereafter, EBs were washed with phosphate/citrate acid buffer (pH 7.4) followed by filtering to remove any large clump. Then the cells were resuspended and washed in antibody stain solution (0.15 Triton X and 1% BSA in 1× PBS) twice, and resuspended in 100 μl of antibody staining solution. The cells were stained with 5 μl of anti‐BrdU‐FITC antibody (eBiosciences 11‐5071‐42) for 30 min at RT. BrdU incorporation data were collected in a ZE5 flow cytometer and analyzed using FlowJo Software.

### Alkaline phosphatase staining

Alkaline phosphatase staining was measured using the Alkaline Phosphatase Detection kit (Stemgent) according to standard protocols from the manufacturer. Brightfield images were obtained using a Zeiss microscope and analyzed by ZEN lite software.

### Immunocytochemistry analysis

Cells were fixed in 4% formaldehyde (Sigma‐Aldrich) and permeabilized with 0.25% Triton X‐100 (Sigma‐Aldrich). Following permeabilization, cells were washed twice with PBS and blocked with 10% goat serum (Invitrogen) and bovine serum albumin (Hyclone) at RT for 1 h. Cells were then stained with primary antibodies overnight at 4°C at corresponding dilution as previously indicated, and secondary staining was performed using corresponding secondary antibodies. DAPI was used for DNA nuclear staining. Images were acquired using a Nikon microscope (ZEN lite software) and analyzed using ImageJ. The total fluorescence signal was measured using a threshold setting.

### CHX treatment and proteasome inhibition

Mouse ESCs were seeded at a density of 1 × 10^5^ cells/ml in 6‐well plate and after 24 h; cells were treated with cycloheximide (CHX) at a final concentration of 50 μg/ml for 0, 3, 6, and 9 h, respectively. For proteasome inhibition, cells were incubated with MG‐132 at a final concentration of 10 μg/ml for 0, 4, and 8 h, respectively. Cells were harvested for whole cell extracts at indicated time points and subjected to immunoblotting. Blots were quantified using Image Lab software.

### RT‐qPCR

The extraction of total RNA was performed using the RNeasy Plus Mini Kit (Qiagen). One microgram of total RNA was used for complementary DNA synthesis using RevertAid First‐Strand cDNA Synthesis kit (Invitrogen) as per the manufacturer’s instructions. RT‐qPCR was performed in triplicate using the PowerUp SYBR Green Mix (Thermo Fisher Scientific) and the primers listed in Appendix Table S1 on a QuantStudio Real‐time PCR instrument (Applied Biosystems). *β‐Actin* levels were used to normalize input amounts. After each round of amplification, melting curve analysis was carried out in order to confirm the PCR product specificity. Ct values obtained for each gene were analyzed using the ΔΔCt method from at least three biological replicates.

### RNA‐Seq and differential gene expression analysis

RNA‐seq library preparation was carried out at Novogene facilities (https://en.novogene.com/) and sequenced using Illumina HiSeq 2500 platform (Illumina) as 150‐bp pair‐ended reads. FASTQ reads were aligned to the ENSEMBL mouse transcriptome using BWA‐MEM. Then, the calculation of normalized reads per transcript was retrieved by Express (Roberts & Pachter, 2013). Thereafter, DEG was obtained by using a test under the assumption of a negative binomial distribution where the variance is linked to the mean via a locally regressed smooth function of the mean (Anders & Huber, 2010) and *P*‐values were adjusted by estimation of the false discovery rate for multiple hypothesis. We only considered the genes with TPM > 0 in at least 2 of the samples. FDR value was calculated with the Benjamini–Hochberg correction.

### AS and data analysis

SUPPA2 was used as a pipeline for AS analysis (Patro *et al*, 2017). Briefly, FASTQ reads from RNA‐seq experiment were aligned and pseudo quantified to mouse genome (mm10) using Salmon (Trincado *et al*, 2018). Splicing events in the mouse genome were obtained using a specific SUPPA2 script from a mouse GTF file. Thereafter, percent splicing inclusion (PSI) values for each event were obtained, and the differential PSI values for each condition were calculated along with a *P*‐value for each event.

### RT‐PCR

Total RNA was extracted using the RNeasy Mini Kit (Qiagen). Two micrograms of total RNA were reverse transcribed using the RevertAid First Strand cDNA Synthesis kit (Invitrogen). RT‐PCR was performed using the DreamTaq Green Master mix (Thermo Fisher Scientific). The specific primers used for AS are listed in Appendix Table S1. The ratios between splice variants were determined by densitometry using ImageJ software. The percentage of inclusion was calculated by dividing the area of the inclusion band by the combined area of both inclusion and exclusion bands and multiplied by 100.

### Gene Ontology (GO) analysis

Gene ontology (GO) analysis was performed using the web tool The Database for Annotation, Visualization and Integrated Discovery (DAVID) (http://david.abcc.ncifcrf.gov/).

### Heat maps

Heat maps were made using *Z*‐score, which was calculated as following: Z = (X‐Xav)/Xsd, where X is the log_2_‐transformed expression level for a given gene in a specific sample, Xav is the mean of log_2_‐transformed expression values for that gene in all samples, and Xsd is the standard deviation of log_2_‐transformed expression values for that gene across all samples.

### *In* *vitro* RNA immunoprecipitation (RIP) and data analysis

cDNA of *Zfp207* isoform 3 from mouse ESCs was generated (RevertAid^®^ First Strand cDNA Synthesis Kit) and cloned into a HaloTag backbone as a ZFP207‐Halo fusion (primers are described in Appendix Table S1). Zfp207‐Halo plasmid was linearized, *in vitro* transcribed (MegaScript T7 Transcription kit, Thermo Fisher #AM1333) and *in vitro* translated (Wheat Germ *in vitro* translation kit, Promega #L4380). The *in vitro‐*translated ZFP207‐Halo fusion product was incubated with total RNA from mouse ESCs. Prior to pull‐down mouse ESCs, total RNA was chemically fragmented at 95°C for 4 min in 100 mM of Tris‐HCl and 10 mM of MgCl_2_ resulting in a population of ~50 nt long RNA fragments that can be bound by ZFP207 and cloned subsequently using a small RNA library preparation protocol. After this binding reaction, the RBP–RNA complex was purified and the bound RNA eluted for subsequent Illumina library preparation (NEXTFLEX small RNA library preparation kit v3, PerkinElmer #NOVA‐5132‐06) and paired‐end (40 × 43 cycles) sequenced on an Illumina NextSeq 500. Sequencing reads were trimmed, adapters and ribosomal RNA reads removed and aligned against the reference genome GRCm38.p6 retrieved from GENCODE (https://www.gencodegenes.org/mouse/) using HISAT2 v2.1.0 with default parameters. Only uniquely mapping reads were further considered. PCR duplicate reads were removed. The libraries of the two replicates, HaloTag and input were read count normalized. The input RNA size was 50 nt in length. Under this consideration, we expect that fragments bound by ZFP207 will result in an enrichment of alignable reads with respect to HaloTag and Input control libraries. This peak enrichment is an indication of binding events and binding sites. Binding events (peaks) were called by using MACS2 v2.2.6 (https://github.com/macs3‐project/MACS/wiki/Advanced:‐Call‐peaks‐using‐MACS2‐subcommands). Genomic regions enriched over Halo and Input signals are kept and further filtered at a FDR ≤ 0.01, a fold change (FC) enrichment FC ≥ 3 (Halo) and FC ≥ 2 (Input) and RPM ≥ 2 in both replicates. Only consensus peaks between the two replicates were considered further. Genomic features (R Genomic Features package (v1.36.4) and R Genomic Ranges package (v1.36.1)) were used to annotate the peaks, and pathway enrichment was conducted using R ClusterProfiler (v3.12.0) to compute the significance of the enrichments and org. Mm.eg.db (v3.8.2) to retrieve Entrez IDs required by ClusterProfiler using the entire murine transcriptome as a background model. Each peak has a reported Binding Score computed as the base 2 logarithm of the product between the log_2_RPM and the mean enrichment over the two negative controls (HaloTag and Input). For GO, each term was ranked by the sum of all binding scores of all peaks present in this pathway. Circle diameter represents the number of genes bound by ZFP207 in that specific pathway.

### *In* *vivo* RNA immunoprecipitation (RIP)

Mouse ESCs were transfected with 15 μg of pCMV‐3XFLAG ZFP207 or empty pCMV‐3XFLAG using Lipofectamine LTX reagent with Plus reagent (Invitrogen). After 48 h, cells were harvested and lysed with 1× NP lysis buffer (50 mM of Tris‐HCl pH 7.5, 100 mM of NaCl, 0.5% (v/v) NP40, 2 mM of EDTA, 1× Protease inhibitor cocktail (Thermo), and 1× diluted Ribolock RNase inhibitor (Thermo)) with gentle rotation for 30 min at 4°C. The supernatant was separated after centrifugation at 12,000 *g* for 15 min at 4°C. The lysate was precleared with protein A agarose magnetic beads (Pierce) for 30 min at RT with gentle rotation. Thereafter, the lysate was incubated with FLAG antibodies in a ratio of 1 μg of FLAG antibodies to 2 mg of lysate for 1 h at RT with gentle rotation, and subsequently immunoprecipitated with protein A beads for 3 h at 4°C. Beads were washed three times with IP wash buffer (50 mM of Tris‐HCl pH 7.5, 300 mM of NaCl, 0.05% (v/v) NP40), three times with high salt wash buffer (50 mM of HEPES‐KOH pH 7.5, 500 mM of NaCl) and twice with PNK buffer (50 mM of Tris‐HCl pH 7.5, 50 mM of NaCl, 10 mM of MgCl_2_). All washes were carried out at 4°C with gentle rotation for 5 min. One‐tenth of the samples were analyzed by SDS–PAGE in order to check IP efficiency. For RT‐qPCR analysis, proteins were removed by proteinase K digestion, RNA was extracted using Trizol, and RNA was reverse‐transcribed with the SuperScript VILO cDNA synthesis kit (Thermo). RT‐qPCR analysis of the cDNA was performed with specific primers as indicated in Appendix Table S1.

### Subcellular RNA fractionation

Two million cells were harvested and washed twice with PBS. After centrifugation for 10 min at 500 *g*, the pellets were resuspended in 2 packed volumes of cytoplasmic lysis buffer (10 mM of Tris‐HCl pH 7.5, 0.15% NP‐40, 150 mM of NaCl supplemented with proteinase and RNase inhibitors) and mixed well by pipetting up and down 15 times. The mix was then incubated on ice for 10 min and 1.5 times volumes of chilled sucrose buffer (10 mM Tris‐HCl pH 7.5, 150 mM NaCl, 24% sucrose) were added to the lysate. The lysate was then centrifuged at 4°C, 9,500 *g* for 3 min, and the supernatant was collected as cytoplasmic fraction. The nuclear pellet was resuspended in 2 packed volumes of cytoplasmic lysis buffer without NP‐40 and mixed with 1.5 times volumes of chilled sucrose buffer followed by centrifugation at 9,500 *g* 5 min at 4°C. The washed nuclear fraction was resuspended with chilled glycerol buffer (20 mM of Tris‐HCl pH 7.9, 75 mM of NaCl, 0.5 of mM EDTA, and 50% glycerol) followed by addition of equal volume of cold nuclei lysis buffer (10 mM of HEPES pH 7.6, 1 mM of DTT, 7.5 mM of MgCl_2_, 0.2 mM of EDTA, 0.3 M of NaCl, 1 M of Urea, 1% NP‐40 supplemented with proteinase and RNase inhibitor). The mixture was vortexed twice for 5 s each and incubated on ice for 2 min followed by centrifugation at high speed for 5 min. The supernatant was collected as a nuclear fraction. Total RNA from cytosolic and nuclear fraction was extracted using the RNeasy Mini Kit (Qiagen).

### RNA FISH

Cells were grown on microscope coverslips placed in tissue culture dishes. After washing twice with PBS, cells were fixed in 4% paraformaldehyde (Sigma‐Aldrich) and permeabilized with 70% Ethanol for 2 h at 4°C. Following permeabilization, cells were washed twice with 25% of RNA wash buffer (25% formamide, 2× SSC) for 5 min at RT. Cells were stained with hybridization mix (10% dextran sulfate, 25% formamide, 2 × SSC, 1 mg/ml *E. coli* tRNA, 0.02% BSA, 10 mM vanadyl–ribonucleoside complex) and 2 µM 5´Cys3‐oligo dT(50) probe for 24 h at 30°C. The following day, coverslips were washed with 25% of RNA wash buffer for 60 min at 30°C and incubated with 20 ng/ml DAPI (RNAse‐free) in 25% RNA wash 30 min at 30°C. The coverslips were then rinsed with 2 × SSC buffer followed by RNA equilibration buffer (10 mM of Tris‐HCl pH 7.5, 2 × SSC and 0.4% glucose). Thereafter, coverslips were mounted using an anti‐bleach buffer (10 mM trolox, 37 ng/μl glucose oxidase, catalase in RNA equilibration buffer). Images were acquired using a Nikon microscope (ZEN lite software) and analyzed using ImageJ. For quantifying nuclear poly(A)^+^ RNA signals (N), images containing at least 30 nuclei were only considered. DAPI staining was masked and overlaid on top of the 5′Cys3‐oligo d(T) signal, after that intensity of pixel was measured using analyze particles. Then, the total FISH signal was measured as fluorescence using a threshold setting. And the cytosolic signal was measured as subtracting the nuclear signal from the total signal. All the images were exposed to the same magnitude of gain.

### Statistical analysis

Data are shown as mean ± SEM. GraphPad Prism version 8.0.0 for Windows was used to perform the statistical analysis (GraphPad Software, La Jolla California USA, www.graphpad.com). The significance was determined using the student's *t*‐test, the ordinary one‐way ANOVA, two‐way ANOVA and ratio paired *t*‐test where indicated including correction for multiple comparison. Probability values of *P < 0.05, **P < 0.01, ***P < 0.001 ****P < 0.0001 were considered as statistically significant.

## Author contributions

FA conceived and designed the study. SM, DPB. PG, DM‐S, and CM‐G performed experiments, IA and CK performed *in vitro* RIP analysis, A‐CR, DZ, and D‐FL performed bioinformatics analysis. FA wrote the manuscript. All authors reviewed and edited the manuscript.

## Supporting information



Appendix

Expanded View Figures PDF

Dataset EV1

Dataset EV2

Dataset EV3

Source Data for Expanded View and Appendix

Review Process File

Source Data for Figure 1

## Data Availability

All next‐generation sequencing data can be publicly accessed in ArrayExpress webserver (E‐MTAB‐10108, http://www.ebi.ac.uk/arrayexpress/experiments/E‐MTAB‐10108/ and E‐MTAB‐10113, http://www.ebi.ac.uk/arrayexpress/experiments/E‐MTAB‐10113/).
