# Supplementary material for: ZFP207 sustains pluripotency by coordinating OCT4 stability, alternative splicing and RNA export
Source: EMBO Rep. 2022 Jan 17;23(3):e53191. doi: 10.15252/embr.202153191 (PMC8892232; doi:10.15252/embr.202153191)
Supplement: Supplementary file 1 — Appendix [file EMBR-23-e53191-s006.docx]

APPENDIX

***ZFP207 regulates post-transcriptional mechanisms to sustain pluripotency***

Sandhya Malla^1,2,3^†, Devi Prasad Bhattarai^1,2,3^†, Paula Groza^2,3^, Dario Melguizo-Sanchis^1,3^, Ionut Atanasoai^4^, Carlos Martinez-Gamero^2,3^, Ángel-Carlos Román^5^, Dandan Zhu^6^, Dung-Fang Lee^6,7,8,9^, Claudia Kutter^4^, Francesca Aguilo^1,2,3*^

^*^Correspondence to: [francesca.aguilo@umu.se](mailto:francesca.aguilo@umu.se)

†These authors contributed equally to this work

**Appendix Table of Contents**

**Appendix Figures:**

**Appendix Figure S1.** Generation of *Zfp207* KO mouse ESC lines, related to Figure 1, p. 2.

**Appendix Figure S2.** Ablation of *Zfp207* disrupts differentiation, related to Figure 2, p. 3.

**Appendix Figure S3.** Silencing of *Zfp207* leads to a blockade at early ectodermal differentiation, related to Figure 3, p.4.

**Appendix Figure S4.** Identification of ZFP207 targets, related to Figure 4, p. 5.

**Appendix Figure S5.** ZFP207 binding profile to mRNAs, related to Figure 6, p. 6.

**Appendix Table:**

**Appendix Table S1.** Primers used in this study, pp. 7-9.

**Appendix Figure S1**

**Appendix Figure S1. Generation of *Zfp207* KO mouse ESC lines, related to Figure 1.**

A. Diagram of sgRNA1 and sgRNA2 targeting the exons 3 and 9 of the *Zfp207* gene, respectively (top) and corresponding sites of its encoded protein (bottom). The sgRNAs and PAM sequences are marked in red and blue, respectively.

B. Detailed information on the strategies used for KO generation, number of clones picked, and the number of homozygous clones obtained.

C. WB analysis of selected clones from two different strategies (sgRNA #1 and a combination of sgRNA #1 and #2) used to evaluate the expression of ZFP207. WT: Wild-type; C1: clone 1; EV: empty vector; and C2: clone 2.

D-E. Sanger sequencing analysis (upper panel) and chromatogram (lower panel) of clones (D) C1 and (E) C2. Insertion is indicated in red while deletions are represented in dashes and in black arrows in the chromatogram.

**Appendix Figure S2**

**Appendix Figure S2. Ablation of *Zfp207* disrupts differentiation, related to Figure 2.**

A. Flow cytometric profile of BrdU incorporation of shScr, sh1 and sh2 along the course of EB differentiation.

B-D. (B) TUNEL (green) staining and (C) quantification of the signal in shScr, sh1 and sh2 at days 4, 6, and 8 of EB differentiation. Nuclei were counterstained with DAPI. Scale bar, 20 µM. (D) Percentage of live (Annexin V-) and apoptotic cells (Annexin V+) in sh1 and sh2 compared to shScr EBs at day 8 of differentiation.

E. RT-qPCR of the neural-associated markers (*Pax6, Sox11, and Nestin*) at day 4 of EB differentiation in shScr and *Zfp207*-depleted ESCs (sh1 and sh2). mRNA levels are relative to the expression of shScr.

Data information: data are presented as mean ± SEM or representative images of *n* ≥ 3 independent biological experiments. ***P* < 0.01, ns = no significant difference. C and D: unpaired Student’s *t*-test; E: Ratio paired t test.

**Appendix Figure S3**

**Appendix Figure S3. Silencing of *Zfp207* leads to a blockade at early ectodermal differentiation, related to Figure 3.**

A. Immunostaining of NESTIN (green) and TUJ1 (red) in shScr, sh1 and sh2 at days 5 (left panel) and 6 (right panel) of neuroectodermal differentiation. Nuclei were counterstained with DAPI. Scale bar, 20 µM.

B-C. (B) TUNEL (green) and (C) CASPASE 3 (red) staining in shScr, sh1 and sh2 at day 5 of neuroectodermal differentiation. Nuclei were counterstained with DAPI. Scale bar, 20 µM.

D. RT-qPCR of the neural-associated markers (*Pax6, Sox11, Nestin, and Tuj1*) at day 4 and day 5 of neural directed differentiation in shScr and *Zfp207*-depleted ESCs (sh1 and sh2). mRNA levels are relative to the expression of shScr at day 0.

Data information: data are presented as mean ± SEM or representative images of *n* ≥ 3 independent biological experiments. **P* < 0.05, ***P* < 0.01, ****P* < 0.001, ns = no significant difference. D: unpaired Student’s *t*-test.

**Appendix Figure S4**

**Appendix Figure S4. Identification of ZFP207 targets, related to Figure 4.**

A. GO analysis of molecular functions associated with common down-regulated genes in *Zfp207*-depleted ESCs (sh1 and sh2) compared to shScr.

B. RT-qPCR analysis of mitotic sister chromatid segregation related genes (*Cdca8* and *Cep57l1*) in shScr, sh1 and sh2 ESCs. Data are presented as mean ± SEM of *n* ≥ 3 independent biological experiments. **P* < 0.05, ****P* < 0.001; unpaired Student’s *t*-test.

C, D. (C) GO analysis of molecular functions associated with common up-regulated genes and (D) of biological processes associated with poly(A)+ RNA binding up-regulated genes in *Zfp207*-depleted ESCs (sh1 and sh2) compared to shScr.

**Appendix Figure S5**

**Appendix Figure S5. ZFP207 binding profile to mRNAs, related to Figure 6.**

A. *In vitro* RIP-seq binding profile for ZFP207 at *Marf1*, *Sae1*, and *Nsd1* genes. Values on the y axis represent a sequencing depth normalized and HaloTag subtracted read-count. Both replicates (Rep1 and Rep2; red) and the input (grey) are shown.

B, C. (B) Mouse ESCs expressing either pCMV-3XFLAG (empty vector) or pCMV-3XFLAG-ZFP207 were harvested and subjected to RIP with FLAG antibodies, followed by immunoblotting with FLAG and β-ACTIN antibodies, respectively. Percentage of input is 10%. (C) RIP-qPCR for *Klf4*, *Esrrb*, *Igf2bp1*, *Fxr1*, and *Sae1*. *GAPDH* and *β-Actin* were used as a negative control. Enrichment of FLAG-ZFP207 was calculated over FLAG-empty. Data are presented as mean ± SEM or representative images of *n* ≥ 3 independent biological experiments. **P* < 0.05, ***P* < 0.01, *****P* < 0.0001; unpaired Student’s *t*-test

D, E. (D) Pie chart depicting ZFP207 binding sites (black lines) at the Core, PRC and Myc modules and (E) its average binding score.

**Appendix Table S1. Primers used in this study.**

| **RT-PCR** | | |
| --- | --- | --- |
| **Gene** | **Forward Sequence 5'-3'** | **Reverse Sequence 5'-3'** |
| *Zfp207* | AACTTCCTTTCAGCCACAGC | AGGAACACCTGGCATCAATG |
| *Pou5f1* | AGTTGGCGTGGAGACTTTGC | CAGGGCTTTCATGTCCTGG |
| *β-Actin* | ACCAACTGGGACGACATGGAGAAG | TACGACCAGAGGCATACAGGGACA |
| *Sox11* | ACGACCTCATGTTCGACCTGAGCT | CACCAGCGACAGGGACAGGTTC |
| *Nestin* | CTCGGGAGAGTCGCTTAGAG | ACATCTTGAGGTGTGCCAGT |
| *Tuj1* | GTCTCTAGCCGCGTGAAGTC | CATCGCTGATGACCTCCCAG |
| *Sox1* | GTGACATCTGCCCCCATC | GAGGCCAGTCTGGTGTCAG |
| *Itga1* | ATGACGCTCTGCCAAACTCA | TGTTGTACGCACTGTCTCCC |
| *Efna5* | GGAGATGTTGACGCTGCTCT | CGTAGCGCTCAGTCTTGTCT |
| *Id1* | CGCATTTTCATCGTGCCTCC | TCAGCGACACAAGATGCGAT |
| *Fgf18* | GGACCAGTGGGAAGCACATT | CGAGCTTGCCTTTTCGGTTC |
| *Sox17* | GCCGAGCCAAAGCGG | GTCAACGCCTTCCAAGACTTG |
| *Foxa2* | CCCTACGCCAACATGAACTCG | GTTCTGCCGGTAGAAAGGGA |
| *T* | GCTTCAAGGAGCTAACTAACGAG | CCAGCAAGAAAGAGTACATGGC |
| *Msx1* | TCATGGCCGATCACAGGAAG | CGACTGAGAAATGGCCGAGA |
| *Pax6* | ACACGTACAGTGCTTTGCCA | ACACGTACAGTGCTTTGCCA |
| *Nanog* | TCTTCCTGGTCCCCACAGTTT | GCAAGAATAGTTCTCGGGATGAA |
| *Ngfr* | AGGGCATTTGTGGAGGGATG | TGGATTATGGGGTGGGTCCT |
| *Nde1* | GGTGATTTTAAGCTGCTGGCT | GTGCACAGACATGAAAGGACG |
| *Itga1* | CTGAGGACAGGAGACGTGGT | GGGCTCTTCACTGTCACTTG |
| *Efna5* | TGCAATCCCAGACAACGGAA | TCGAAAACACGATCATGAACACC |
| *Id1* | GAGTCTGAAGTCGGGACCAC | GGAACACATGCCGCCTCG |
| *Fgf18* | GAGGACGGGGACAAGTATGC | TAGTACCATCAGGCTTCCCCA |
| *Son* | AACCTAAAGCCTGCACCTCC | GCAATCTGTTTGCATTTCTCAGTT |
| *Ybx1* | GGGGTAGAGAAGCTTTGTGGG | AAGTTGATGGACTTGGCGGA |
| *Mbnl2* | GCGGCTCGCAGAGTACAATA | AAGAACATCTGGCAGGACCG |
| *Ptbp1* | CAGCGGGTGAAGATCCTGTT | GTAATGCGCACTGACTTCCC |
| *Srsf7* | TCAAGATCAGCATCTCCTCGC | TCTCGACCTTGAGCGGGAT |
| *Sf3b3* | TTTGCCTTGAAACCGTTGCTG | CCTGTTGCTTGGTTCCAGAGAAG |
| *Cdca8* | AGGTTTGACTCCCGGGTCTT | TATGGGCACACTGAGGGAGA |
| *Cenpa* | CAGACACCCGATACCTGCC | ACTGCCACGGCTCACATAC |
| *Cep57l1* | GACAGAGCCTGTGAGAAAACTAA | GCTCTAGCCTGGGAAGTTGTT |
| *Ubp1* | GCCTAAAGGTGCAGACAGAAAA | TCTGTGAGAATGGTGGTGTCG |
| *Marf1* | GGTGTCGGAATGTGAGCCAA | TCCCTTCCATCACATAGCCAG |
| *Essrb* | ACAGCACCAAAGAGAAGCAC | AAGTCATTCCAGCCACAACG |
| *Klf4* | AGAAGAAGGATCTCGGGCAATC | TACCTGAGCCCCAAAGTCAAC |
| *Igf2bp1* | TCTGGCAGAATGGGTTGATAGG | GTCTAACAGTCACAAGCAGCAG |
| *Fxr1* | TCAAGAGCAAATGACCAAGAGC | TTACCTCGCCTTTCATCATCCG |
| *Gapdh* | CTCCCACTCTTCCACCTTCG | GCCTCTCTTGCTCAGTGTCC |
| *Srfs11* | CTCCAGCAGGCATGGATTGT | TAGGTCACCGTGTCAGAAAGT |

| **Primers for cloning shRNAs** | | |
| --- | --- | --- |
| **Target Gene** | **Plasmid** | **Sequence 5'-3'** |
| Scramble | pLKO.1-puro-shRNA_Scramble | CAACAAGATGAAGAGCACCAA |
| *Zfp207* | pLKO.1-Puro-shRNA-*Zfp207*_1 | GAAGGGCACAGTTACCTAAAT |
|  | pLKO.1-Puro-shRNA-*Zfp207*_2 | GGACAAACTCCAATTGGTAAT |
| *Srfs11* | pLKO.1-puro-shRNA_*Srfs11_1* | GCAGAGATGAAAGGGAACGAT |
|  | pLKO.1-puro-shRNA_*Srfs11_2* | CCCTGATTTCTGCCGCTATTG |

| **sgRNA** | | |
| --- | --- | --- |
| **Target gene** | **Plasmid** | **Sequence 5'-3'** |
| *Zfp207* | pSpCas9(BB)-2A-Puro-sgRNA-*Zfp207*_1 | CACCGCAGTACCAAATGCAATACCT |
|  | pSpCas9(BB)-2A-Puro-sgRNA-*Zfp207*_2 | CACCGATCGTTACTTTATAGGCTC |

| **CRISPR Cas9 validation primers** | | |
| --- | --- | --- |
| **Gene** | **Primer name** | **Sequence 5'-3'** |
| *Zfp207* | P1 | GCAAGAGCTTTGGCCCAGTT |
|  | P2 | AGGAAGAGGACTCAGCAGGTAT |
|  | P3 | CAGCCCAGTATTTTTGGATATTGA |
|  | P4 | ACTTCTCCAATGGGCAGGTT |

| **RT-PCR** | | |
| --- | --- | --- |
| **Gene** | **Forward 5'-3'** | **Reverse 5'-3'** |
| *Mta1*AS1 | GTTACAGACAGCCAACGGGA | GTTACAGACAGCCAACGGGA |
| *Mta1* AS2 | CATCCAGTGTGCTCAGCAGT | CCTGGTCTGTCCATGGTTTG |
| *Nasp* | GAGTGTTGGGAAATGCCTTAGA | AGCCTTGTCATTTTCTCTGTCC |
| *Hnrnpa1 AS1* | CGTGGTGGTGGTGGATATG | TTGGTTCCGTGGTTTAGCA |
| *Tbx3* | GAACCCGAAGAAGACGTAGAAG | GGCAGTAACGGCGATGAA |
| *Ubp1* | AAGGCTGGAAGTGGAATCG | GGCTTGGATTTGTCCTTGTG |
| *Eif4H* | GTGGCTTTGGATTCAGGAAA | GGTCTCTGTGCTCGTTCCT |
| *Fxr1* | ATACAGAATCCGATCAGACTGCA | TTTCTTCTCCCAGAGTACGCG |
| *Myef2* | TGGAGGCATTGGAATGGGAC | ACCAAAGCCTGCCATGCTAT |
| *Vegfa* | CAAACCTCACCAAAGCCAGC | CCACAAAGCATGCCATGTCC |
| *Zfp207* | CCTCCAATGACTCAAGCACA | ATGTAGGCTTTGGGGGTTCT |

| **Zfp207 cloning** | |
| --- | --- |
| **Name of primer** | **Sequence 5´-3´** |
| CDS_*Zfp207*_1_HindIII_FW | TAAGCAAAGCTTATGGGTCGCAAGAAGAAGAA |
| CDS_*Zfp207*_1_BglII_RV | TGCTTAAGATCTTCAGTAACGGCCACCTTGCG |
| ZFP207-Halo fusion_FW | CGAATTCCTACCGCGGATCTATGGGTCGCAAGAAGAAGAAGC |
| ZFP207-Halo fusion_RV | ACAGATCCTCAGTGGTTGGCCCGTAACGGCCACCTTGCGACATTAC |
| Esp3I-FW-207CDS | GTAAACGTCTCCGCCTGACCATGGGTCGCAAGAAGAAGAA |
| XhoI-RV-207CDS | TGCTTACTCGAGTCAGTAACGGCCACCTTGCG |
| Mutagenesis | AGAGAAATCTTCCTCGACCAGGCCAGACGCCCATCGGCAACCCACCAGTTGGACCAATTGGGG |
