# Supplementary material for: ZFP207 sustains pluripotency by coordinating OCT4 stability, alternative splicing and RNA export
Source: EMBO Rep. 2022 Jan 17;23(3):e53191. doi: 10.15252/embr.202153191 (PMC8892232; doi:10.15252/embr.202153191)
Supplement: Supplementary file 2 — Expanded View Figures PDF [file EMBR-23-e53191-s003.pdf]

Expanded View Figures

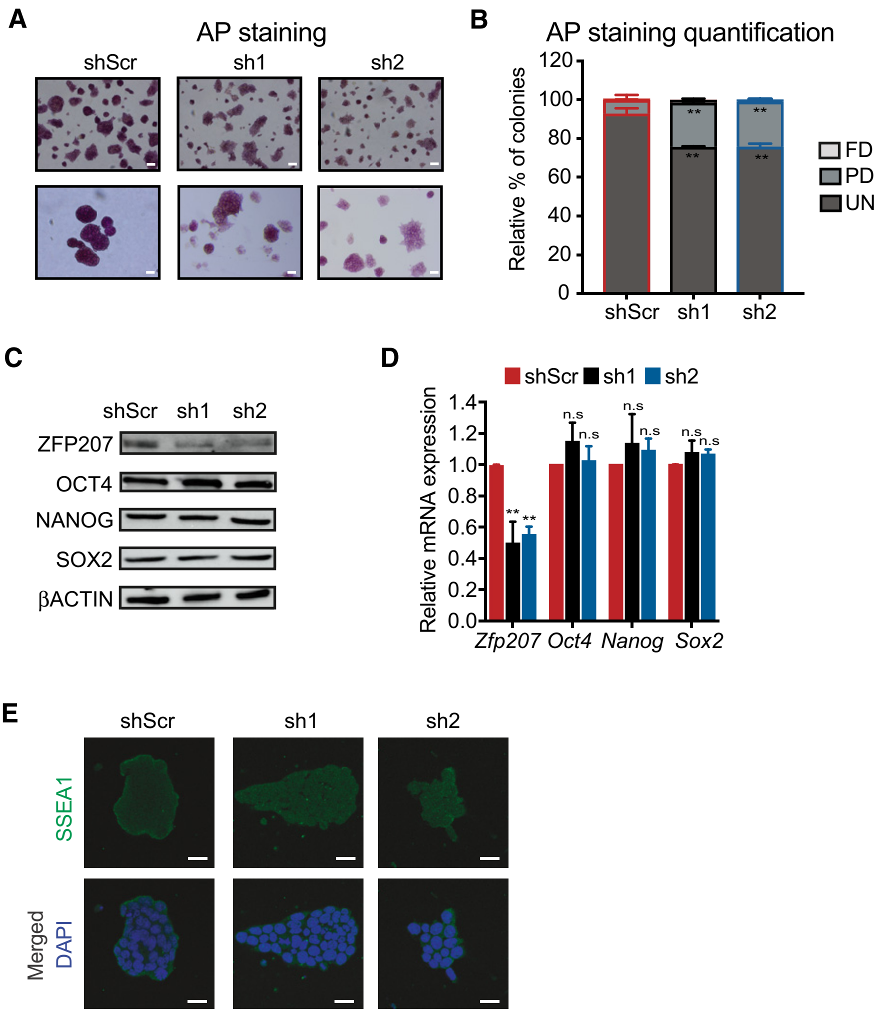

Figure EV1. ZFP207 is dispensable in mouse ESCs maintained in ground state pluripotency.

A, B (A) AP staining of shScr and *Zfp207*-depleted (sh1 and sh2) mouse ESCs cultured in 2iL. Scale bars, 20 μM. (B) Percentage of fully differentiated (FD), partially differentiated (PD) and undifferentiated (UN) ESC colonies in shScr, sh1 and sh2 cultured in 2iL.

C, D (C) Western blot of ZFP207, OCT4, NANOG, and SOX2 and (D) RT-qPCR analysis of *Zfp207*,*Oct4*, *Nanog*, and *Sox2* in shScr, sh1, and sh2 ESCs cultured in 2iL; data are relative to shScr.

E Immunofluorescence analysis of SSEA1 in shScr, sh1, and sh2 ESCs cultured in 2iL. DAPI was used as the nuclear marker. Scale bars, 20 μm.

Data information: Data are presented as mean ± SEM or representative images of $n \geq 3$ independent biological experiments. $^{**}P < 0.01$, n.s = no significant difference (shScr versus sh1 or sh2). B: unpaired Student's *t*-test; D: Ordinary one-way ANOVA.
Source data are available online for this figure.

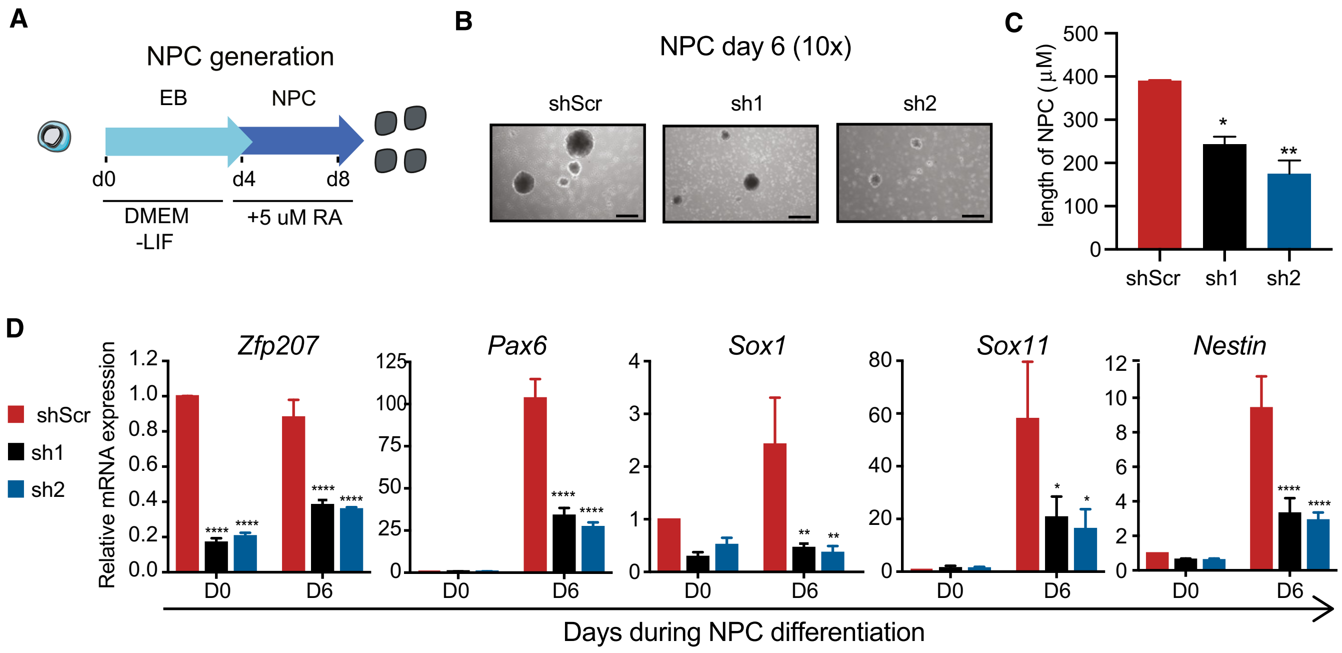

Figure EV2. ***Zfp207*** **knockdown ESCs cannot generate NPCs.**

A Schematic diagram for neural progenitor cells (NPC) generation. Retinoic acid (RA) was added after four days of EB culture.

B, C (B) Representative bright-field images (10×) and (C) quantification of NPCs on day 6 of differentiation. Scale bars, 200 μm.

D RT-qPCR of *Zfp207* and neural-associated markers in shScr, sh1 and sh2 along the course of neural progenitor (NPC) differentiation. mRNA levels are relative to shScr at day 0.

Data information: Data are presented as mean ± SEM or representative images of $n \geq 3$ independent biological experiments. *$P < 0.05$, **$P < 0.01$, ****$P < 0.0001$. C: Ordinary one-way ANOVA; D: two-way ANOVA.

© 2022 The Authors

© 2022 The Authors

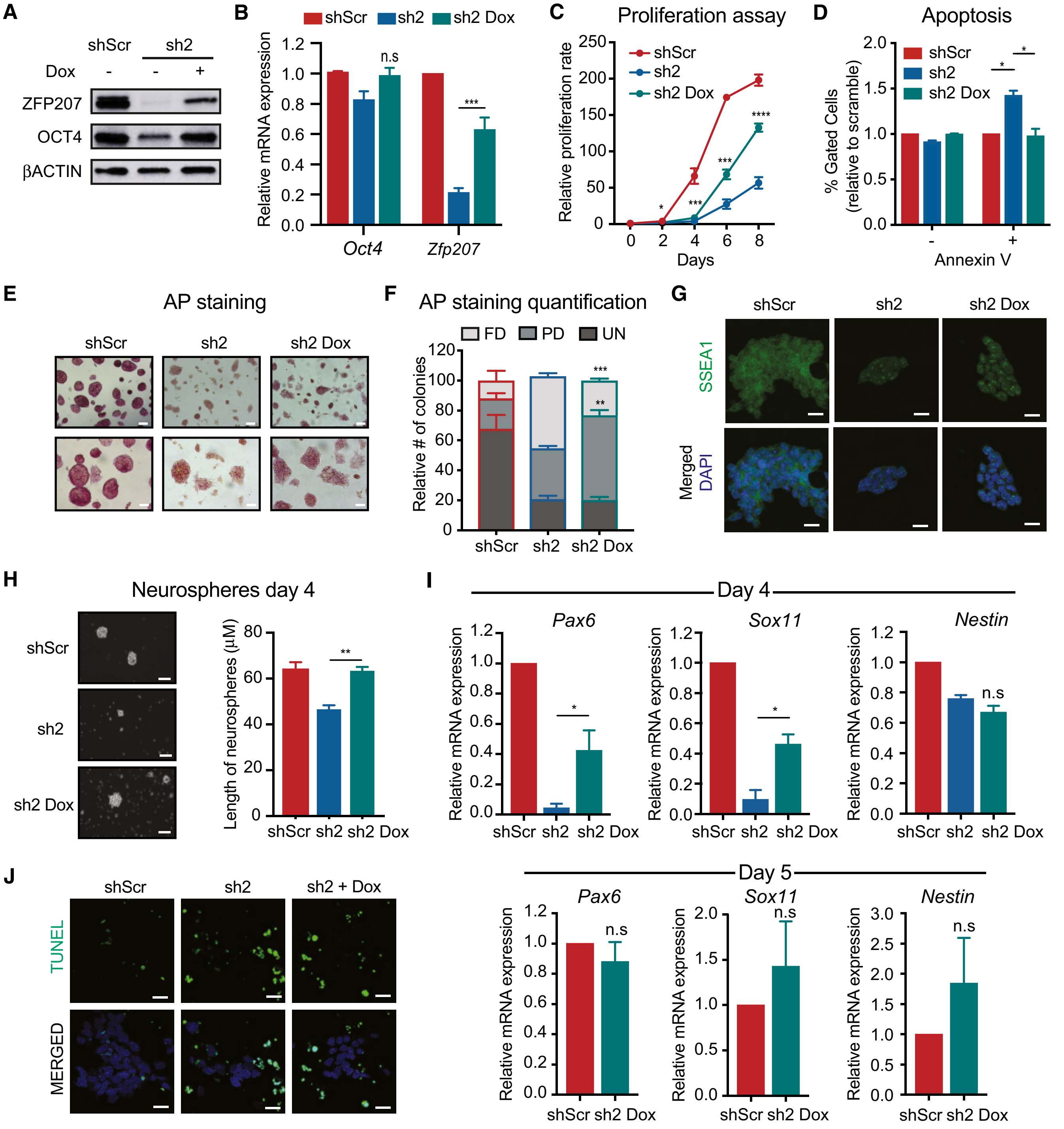

Figure EV3.

© 2022 The Authors

Figure EV3. Re-expression of *Zfp207* in tet(ON)-ZFP207 KD2 rescues the ESC phenotype but it is not sufficient to fully differentiate tet(ON)-ZFP207 KD2 to neurons.

A, B (A) Western blot of ZFP207 and OCT4 and (B) RT-qPCR of *Zfp207 and Oct4* in the tet(ON)-ZFP207 cell line subjected to shScr or sh2 in the absence (−) or presence (+) of doxycycline (Dox) as indicated. mRNA levels are relative to the expression in shScr.

C Relative proliferation rate of tet(ON)-ZFP207 ESCs with shScr and sh2 −/+ Dox assessed over a period of 8 days.

D Percentage of live (Annexin V−) and apoptotic cells (Annexin V+) in tet(ON)-ZFP207 ESCs with shScr and sh2 −/+ Dox.

E, F (E) AP staining of shScr and sh2 −/+ Dox in tet(ON)-ZFP207 ESCs. Scale bar, 50 μM. (F) Percentage of fully differentiated (FD), partially differentiated (PD) and undifferentiated (UN) ESC colonies in shScr and sh2 −/+ Dox treatment in tet(ON)-ZFP207.

G Immunofluorescence analysis of SSEA1 in tet(ON)-ZFP207 ESCs with shScr and sh2 −/+ Dox. DAPI was used as the nuclear marker. Scale bars, 20 μm.

H Representative bright-field images (20x) and quantification of neurospheres on day 4 of differentiation. Scale bars, 200 μm.

I RT-qPCR of neural-associated markers in tet(ON)-ZFP207 ESCs with shScr and sh2 −/+ Dox at day 4 (upper panel) and tet(ON)-ZFP207 ESCs with shScr and sh2 +Dox at day 5 (lower panel). mRNA levels are relative to shScr.

J TUNEL (green) staining in tet(ON)-ZFP207 ESCs with shScr and sh2 +Dox at day 5. Nuclei were counterstained with DAPI. Scale bar, 20 μm.

Data information: Data are presented as mean ± SEM or representative images of $n \geq 3$ independent biological experiments. *$P < 0.05$, **$P < 0.01$, ***$P < 0.001$, ****$P < 0.0001$, ns = no significant difference (sh2 versus sh2 Dox). B, C, D, F and I: unpaired Student's *t*-test.
Source data are available online for this figure.

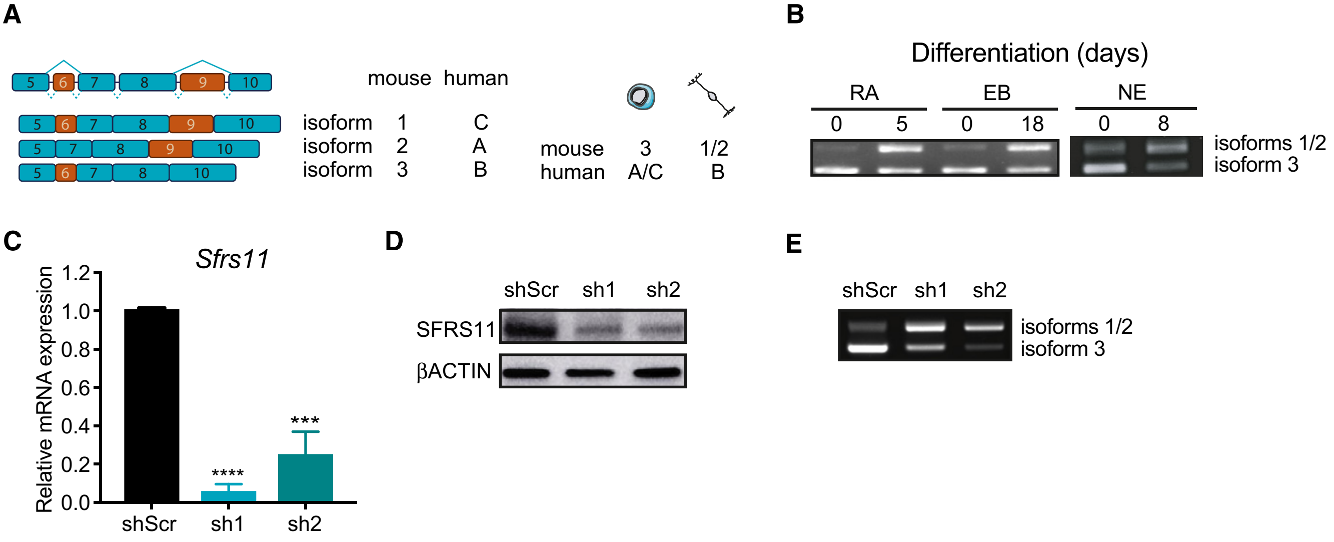

Figure EV4. *Zfp207* undergoes alternative splicing during differentiationz.

A, B (A) Schema showing a segment in transcript variants with alternatively spliced exons of *Zfp207* in mouse and human (left panel). The differential switch of the splice forms between the ESC state and neural-directed differentiation in mouse and human are also depicted (right panel). (B) RT-PCR analysis of the AS forms of *Zfp207* at the indicated time points of retinoic acid (RA) -induced differentiation, embryoid body (EB) generation and neuroectodermal (NE) differentiation of mouse ESCs.

C–E (C) RT-qPCR and (D) western blot to monitor the knockdown efficiency of *Sfrs11* (sh1 and sh2). (E) RT-PCR analysis of the AS forms of *Zfp207* upon depletion of *Sfrs11.*

Data information: Data are presented as mean ± SEM or representative images of $n \geq 3$ independent biological experiments. ***$P < 0.001$, ****$P < 0.0001$ (shScr versus sh1 or sh2). C: unpaired Student's *t*-test.
Source data are available online for this figure.

© 2022 The Authors

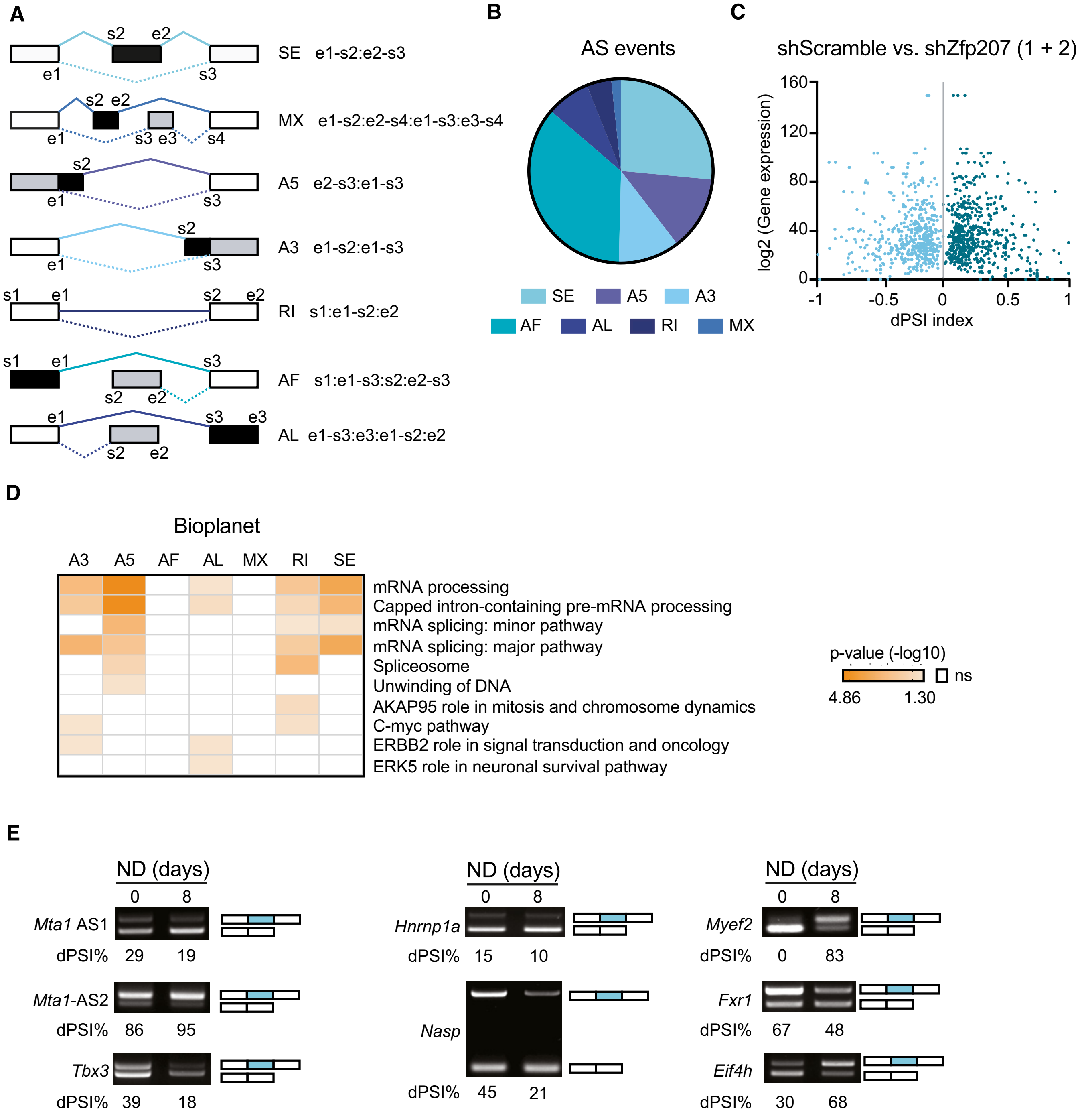

Figure EV5. Alternative splicing switches in *Zfp207* knockdown ESCs are prevalent in differentiated cells.

A Schema depicting the types of AS events: SE, skipped exon; MX, mutually exclusive exons; A5, alternative 5′ splice-site; A3, alternative 3′ splice site; RI, retained intron; AF, alternative first exon; AL, alternative last exon, generated by the software SUPPA2 with the specific coordinates, including start (s) and end (e). The form of the AS event is depicted in black.

B Pie chart showing the AS events types distribution.

C Volcano plot depicting the correlation between gene expression levels and dPSI index resulted from RNA-seq data analysis in control (scShr) and *Zfp207* depleted mouse ESCs (sh1 + sh2). Gene expression levels are presented as log2 transformed values. Differential percent spliced in index (dPSI) range is between −1 and +1.

D Gene ontology enrichment analysis with Bioplanet software for genes undergoing different splicing events upon *Zfp207* depletion (sh1 and sh2).

E Representative RT-PCR analysis of AS events upon neural differentiation in mouse ESCs for *Mta1* (AS1 and AS2), *Tbx3*, *Hnrnp1a*, *Nasp*, *Myef2*, *Fxr1*, and *Eif4H*. The structure of each isoform is indicated (not to scale). Alternative exons are blue. PSI was quantified for each condition.

 © 2022 The Authors
