# Supplementary figures and images for: ZFP207 sustains pluripotency by coordinating OCT4 stability, alternative splicing and RNA export
Source: EMBO Rep. 2022 Jan 17;23(3):e53191. doi: 10.15252/embr.202153191 (PMC8892232; doi:10.15252/embr.202153191)

# Appendix Figure S1C

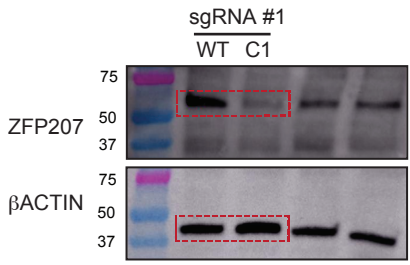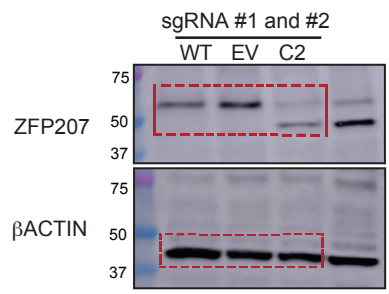

Supplement: Supplementary file 6 — Source Data for Expanded View and Appendix [file EMBR-23-e53191-s004.zip › EV_and_Appendix_Figure_Source_Data/Appendix_FigS1_Source_Data.pdf]

**C**

shScr sh1 sh2

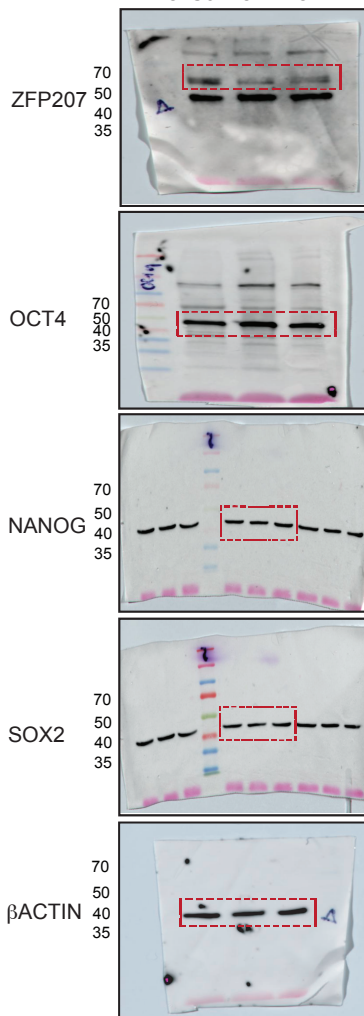

Supplement: Supplementary file 6 — Source Data for Expanded View and Appendix [file EMBR-23-e53191-s004.zip › EV_and_Appendix_Figure_Source_Data/Figure_EV1_Source_Data-sd.pdf]

**D**

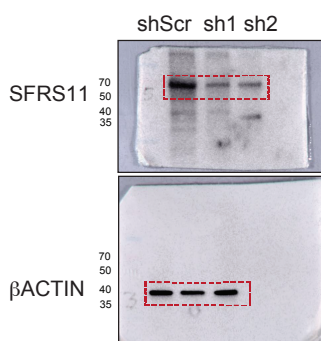

Supplement: Supplementary file 6 — Source Data for Expanded View and Appendix [file EMBR-23-e53191-s004.zip › EV_and_Appendix_Figure_Source_Data/Figure_EV4_Source_Data-sd.pdf]

## Appendix Figure S5B

|             | Input |   | IP |   |
|-------------|-------|---|----|---|
| FLAG-ZFP207 | -     | + | -  | + |
| FLAG-empty  | +     | - | +  | - |

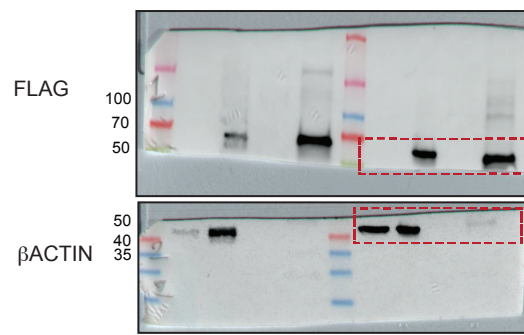

Supplement: Supplementary file 6 — Source Data for Expanded View and Appendix [file EMBR-23-e53191-s004.zip › EV_and_Appendix_Figure_Source_Data/Appendix_FigS5_Source_Data.pdf]

**A**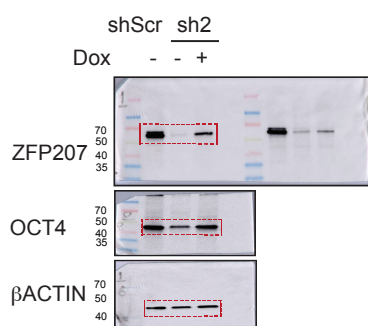

Supplement: Supplementary file 6 — Source Data for Expanded View and Appendix [file EMBR-23-e53191-s004.zip › EV_and_Appendix_Figure_Source_Data/Figure_EV3_Source_Data-sd.pdf]

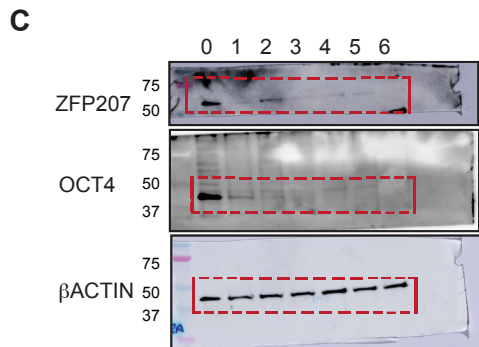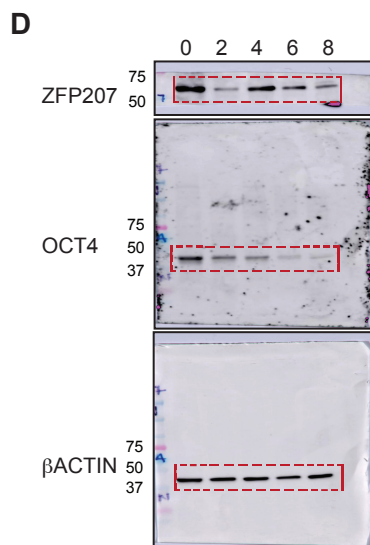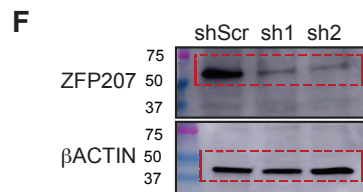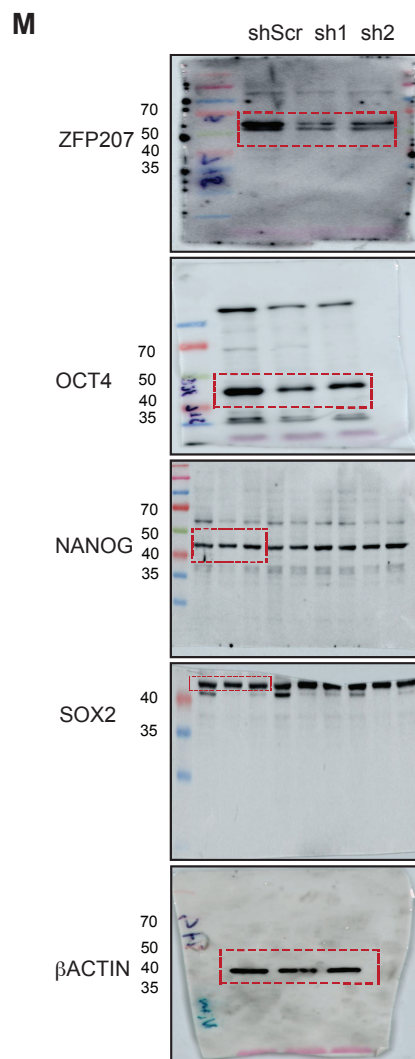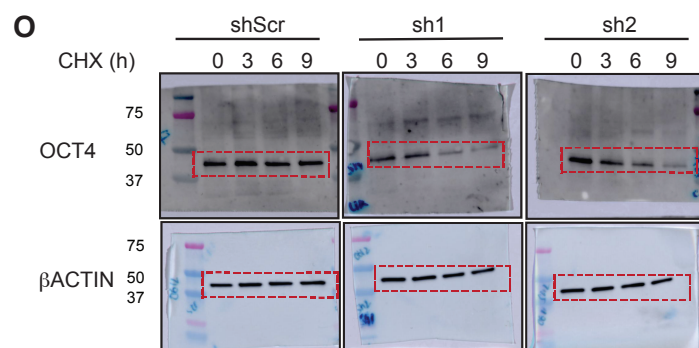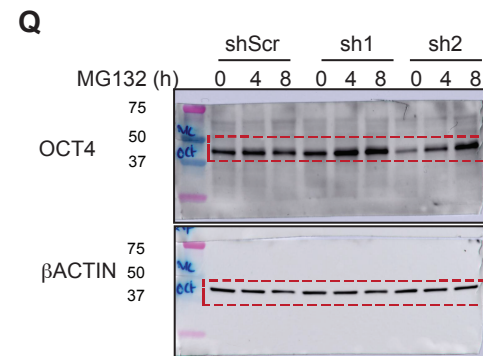

Supplement: Supplementary file 8 — Source Data for Figure 1 [file EMBR-23-e53191-s007.pdf]
